# Exposing the Three-Dimensional Biogeography and Metabolic States of Pathogens in Cystic Fibrosis Sputum via Hydrogel Embedding, Clearing, and rRNA Labeling

**DOI:** 10.1128/mBio.00796-16

**Published:** 2016-09-27

**Authors:** William H. DePas, Ruth Starwalt-Lee, Lindsey Van Sambeek, Sripriya Ravindra Kumar, Viviana Gradinaru, Dianne K. Newman

**Affiliations:** a Division of Biology and Biological Engineering, California Institute of Technology, Pasadena, California, USA; b Division of Geological and Planetary Sciences, California Institute of Technology, Pasadena, California, USA; c Howard Hughes Medical Institute, California Institute of Technology, Pasadena, California, USA

## Abstract

Physiological resistance to antibiotics confounds the treatment of many chronic bacterial infections, motivating researchers to identify novel therapeutic approaches. To do this effectively, an understanding of how microbes survive *in vivo* is needed. Though much can be inferred from bulk approaches to characterizing complex environments, essential information can be lost if spatial organization is not preserved. Here, we introduce a tissue-clearing technique, termed MiPACT, designed to retain and visualize bacteria with associated proteins and nucleic acids *in situ* on various spatial scales. By coupling MiPACT with hybridization chain reaction (HCR) to detect rRNA in sputum samples from cystic fibrosis (CF) patients, we demonstrate its ability to survey thousands of bacteria (or bacterial aggregates) over millimeter scales and quantify aggregation of individual species in polymicrobial communities. By analyzing aggregation patterns of four prominent CF pathogens, *Staphylococcus aureus*, *Pseudomonas aeruginosa*, *Streptococcus* sp., and *Achromobacter xylosoxidans*, we demonstrate a spectrum of aggregation states: from mostly single cells (*A. xylosoxidans*), to medium-sized clusters (*S. aureus*), to a mixture of single cells and large aggregates (*P. aeruginosa* and *Streptococcus* sp.). Furthermore, MiPACT-HCR revealed an intimate interaction between *Streptococcus* sp. and specific host cells. Lastly, by comparing standard rRNA fluorescence *in situ* hybridization signals to those from HCR, we found that different populations of *S. aureus* and *A. xylosoxidans* grow slowly overall yet exhibit growth rate heterogeneity over hundreds of microns. These results demonstrate the utility of MiPACT-HCR to directly capture the spatial organization and metabolic activity of bacteria in complex systems, such as human sputum.

## IMPORTANCE

The advent of metagenomic and metatranscriptomic analyses has improved our understanding of microbial communities by empowering us to identify bacteria, calculate their abundance, and profile gene expression patterns in complex environments. We are still technologically limited, however, in regards to the many questions that bulk measurements cannot answer, specifically in assessing the spatial organization of microbe-microbe and microbe-host interactions. Here, we demonstrate the power of an enhanced optical clearing method, MiPACT, to survey important aspects of bacterial physiology (aggregation, host interactions, and growth rate), *in situ*, with preserved spatial information when coupled to rRNA detection by HCR. Our application of MiPACT-HCR to cystic fibrosis patient sputum revealed species-specific aggregation patterns, yet slow growth characterized the vast majority of bacterial cells regardless of their cell type. More broadly, MiPACT, coupled with fluorescent labeling, promises to advance the direct study of microbial communities in diverse environments, including microbial habitats within mammalian systems.

## INTRODUCTION

Host-microbe interactions are increasingly recognized as drivers of health and disease in many different contexts, from the beneficial human microbiome to deleterious bacterial infections, such as those that chronically infect individuals living with cystic fibrosis (CF) (1–3). In all of these cases, the relationship between microbial and host cells is influenced by the features of the microenvironment, which change over time and can be challenging to measure. Nevertheless, it is essential to characterize the nature of these important associations if we seek to understand and/or control them. Spatial organization is a defining parameter in any environment, and it is likely that by impacting bacterium-bacterium or bacterium-host associations, or by creating gradients of nutrients or toxins that affect bacterial growth rates, spatial organization affects bacterial survival (4). The current toolset for understanding microbial communities associated with animal host environments provides limited spatial information (e.g., thin sectioning) (5–7) or lacks it entirely (bulk measurement of abundance, via metagenomics and transcriptomics) (8–10). Building upon a tissue-embedding and clearing technique, the passive clarity technique (PACT) (11–13), we developed MiPACT (*m*icrobial *i*dentification after PACT) to permit the study of diverse bacterial pathogens residing in cystic fibrosis patient sputum. While PACT preserves spatial and molecular information and allows for efficient clearing as well as protein and transcript labeling via use of fluorescent probes, we incorporated key modifications to ensure (i) stabilization of amorphous sputum samples, (ii) high retention of bacteria, and (iii) efficient labeling of bacterial rRNA via hybridization chain reaction (HCR) (14, 15) and fluorescence *in situ* hybridization (FISH). Though developed in the context of CF, MiPACT-HCR can be readily applied to diverse host-microbe systems.

Patients with CF accumulate obstructive sputum plugs in their lung airways that can harbor an array of opportunistic pathogens (16). Sputum buildup and the resultant chronic infections lead to severe lung damage and eventual respiratory failure (17). CF patients routinely expectorate infected sputum, which provides tractable samples for *in situ* analysis of pathogens (6, 18, 19). Until recently, *Pseudomonas aeruginosa* was the most prevalent pathogen isolated from CF patients, and *P. aeruginosa* colonization is well known to correlate with disease progression in CF (16, 20, 21). Therefore, the majority of studies addressing the biogeography of CF have focused on *P. aeruginosa*. FISH analysis of thin sections of CF lung or smears of CF sputum have revealed that *P. aeruginosa* can exist both as single cells and in large clusters and that *P. aeruginosa* grows more slowly *in situ* than in typical laboratory cultures (6, 7).

While *P. aeruginosa* plays an important role in CF pathogenicity in many patients, other microbes also colonize the CF lung and contribute to exacerbations, or increase disease severity (16). Indeed, culture-independent studies have revealed that individuals harbor a distinct microbial ecosystem whose species composition can vary over time and treatment regimens (10, 22). Though recent studies have attempted to gain a perspective on the distribution of particular clone types as a function of lung geography, these studies have been herculean, requiring microdissection, cultivation, and sequencing of thousands of regional isolates (5, 19, 23). Recognizing the need to study CF pathogens *in situ* to gain information relevant to the design of accurate *in vitro* models, we sought a method that would permit rapid scanning of large spatial areas at various magnifications, as well as one that would permit microbial identification and study at the single-cell level. Here, we describe our usage of MiPACT-HCR to study three important attributes of diverse pathogens in CF sputum: aggregation patterns, bacterium-host interactions, and growth rates.

## RESULTS AND DISCUSSION

We obtained seven sputum samples (numbered 1 to 4 and 5.1, 5.2, and 5.3) with consent from five patients at the Children’s Hospital of Los Angeles (CHLA). Samples 1, 4, 5.2, and 5.3 were collected during an exacerbation, while samples 2, 3, and 5.1 were collected during outpatient well visits. Disease states varied between patients, with patients 1 and 3 having FEV1% (percent forced expiratory volume in 1 s, a measure of lung function) values of 48 and 44 (moderate obstruction), respectively, while the remaining patients had FEV1% values greater than 70 (mild to normal).

**FIG 1:**
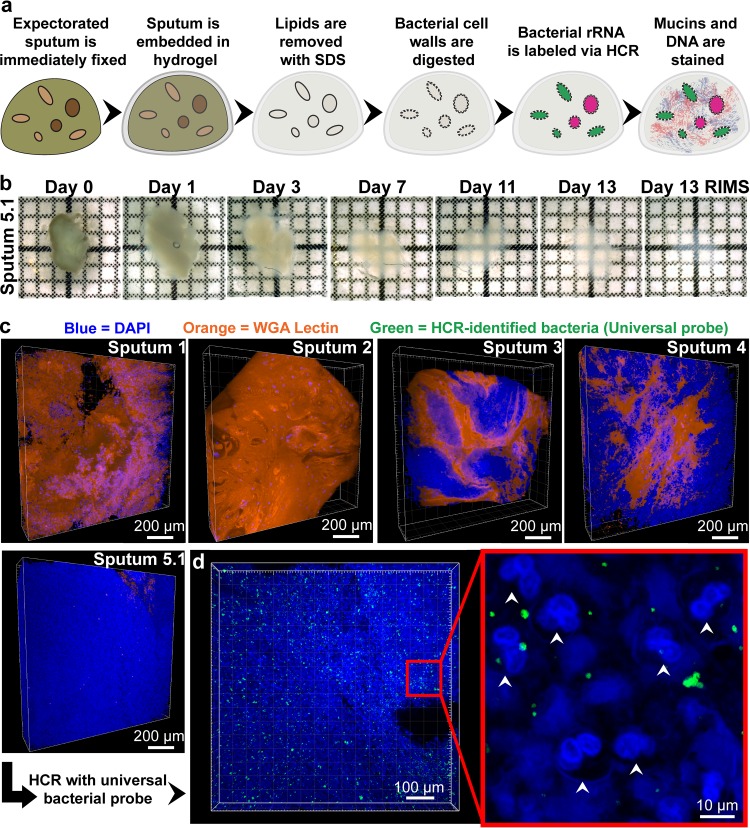
MiPACT-HCR allows visualization of bacteria in cleared sputum samples. (a) Cartoon depicting the process of embedding and clearing sputum for visualization of bacteria via HCR. (b) The clearing process for sputum sample 5.1. Each grid square represents 1 mm^2^. (c) Blend projections of five sputum samples after staining with DAPI (blue) and WGA (orange) from *Z*-stacks acquired with a 10× objective. (d) HCR with a universal bacterial probe (green; EUB338 with B1 hairpins conjugated to AlexaFluor 647) in sputum sample 5.1. The middle panel is a maximum intensity projection acquired with a 10× objective, and the right panel is a single-plane image acquired with a 25× objective. White arrows indicate PMNs.

When fixed in paraformaldehyde (PFA; 4%) and embedded in A_4_P_0_ (4% acrylamide, 0% PFA), sputum completely dissolved during clearing. To provide more structural stability, we replaced acrylamide with 4% 29:1 acrylamide:bis-acrylamide (29A:1B)_4_P_0_, providing additional cross-linking (Fig. 1a). Use of (29A:1B)_4_P_0_ preserved sputum integrity and allowed for clearance in SDS (Fig. 1b). Samples took 3 to 14 days to fully clear (Fig. 1b). Because sputum is composed largely of host-derived DNA and mucins (24), we labeled DNA with 4′,6-diamidino-2-phenylindole (DAPI) and mucins with rhodamine-conjugated lectin (wheat germ agglutinin [WGA]) after clearing to obtain a structural context. Imaging revealed a high degree of compositional variation between samples (Fig. 1c). For example, sputum samples from patients 1 and 2 were composed largely of lectin-stained mucin, with interspersed DAPI-bright host cells. Sputum 5 was composed almost entirely of polymorphonuclear neutrophils (PMNs), consistent with findings that PMNs are a major component of CF patient sputum (25). PMN cell boundaries were outlined by a network of extracellular DNA (Fig. 1d), potentially a result of neutrophil extracellular traps (NETs) (26). While intersample heterogeneity was evident, sampling different regions of a single sputum sample revealed that intrasample composition was relatively homogenous (see Fig. S1 in the supplemental material).

We next verified that the common CF pathogens *P. aeruginosa* and *Staphylococcus aureus* could be retained and visualized under the same embedding and clearing conditions required for retaining sputum integrity. There was no significant loss of DAPI-stained logarithmic- or stationary-phase bacteria after clearing of pure cultures embedded in (29A:1B)_4_P_0_ hydrogel blocks (see Fig. S2a in the supplemental material). FISH staining with saturating probe concentrations of the universal bacterial probe EUB338 after MiPACT (see Fig. S3a in the supplemental material) revealed that the Gram-positive microbe *S. aureus* required treatment with lysostaphin after clearing via SDS, while the Gram-negative *P. aeruginosa* did not require lysozyme treatment (see Fig. S3b). In sputum, autofluorescence makes bacteria, particularly slowly growing cells, difficult to demarcate by FISH (see Fig. S4 in the supplemental material). Therefore, we employed HCR, a FISH amplifying technique which has previously been used to fluorescently label RNA in zebrafish embryos, brain tissue, and environmental microbes (15, 27, 56). HCR entails hybridizing target RNA with a DNA probe that triggers amplification of fluorescently labeled DNA hairpins into polymer chains via a specific initiator region (14, 15). To directly compare FISH and HCR, FISH with a dilabeled AlexaFluor 594 EUB338 probe and HCR with an initiator EUB338 probe and AlexaFluor 594 hairpins were performed separately on stationary-phase cultured cells embedded in (29A:1B)_4_P_0_ and cleared for 5 days. HCR increased the average fluorescence intensity of *P. aeruginosa* cells by ~68-fold and *S. aureus* cells by ~42-fold above levels obtained with FISH.

HCR hybridizations in sputum were optimized such that (i) the EUB338 probe bound and nucleated hairpin polymerization, (ii) samples did not fluoresce when incubated with both NON338, the reverse complement of EUB338, and fluorescent hairpins, and (iii) class/genus-specific probes did not cross-react with other relevant bacteria (see Fig. S5 and S6 in the supplemental material). The Betaproteobacteria probe BET42a, used for *Achromobacter xylosoxidans*, had weak cross-reactivity with *P. aeruginosa* and was therefore not used in *P. aeruginosa* culture-positive samples. Some species-specific probes tested, including those specific for *A. xylosoxidans*, were excluded due to their inability to withstand the stringent hybridization and wash conditions necessary for HCR specificity (see Materials and Methods). Object-based colocalization analysis after HCR multiplexing was performed to further validate HCR specificity. Greater than 90% of objects (discrete HCR-identified cells or aggregates with a size of >4 voxels) in sputum were concurrently identified by using two separate universal probes (see Fig. S6). Moreover, >90% of objects identified by the class/genus-specific probes used in sputum colocalized with EUB338 but not with other class/genus-specific probes (see Fig. S6). HCR allowed multiscale visualization of bacteria; low magnification (e.g., ×10) enabled broad surveying of the sample (Fig. 1d), and increased magnification (e.g., ×25) enabled single-cell resolution and revealed the spatial organization of bacteria and host cells (Fig. 1d).

**FIG 2:**
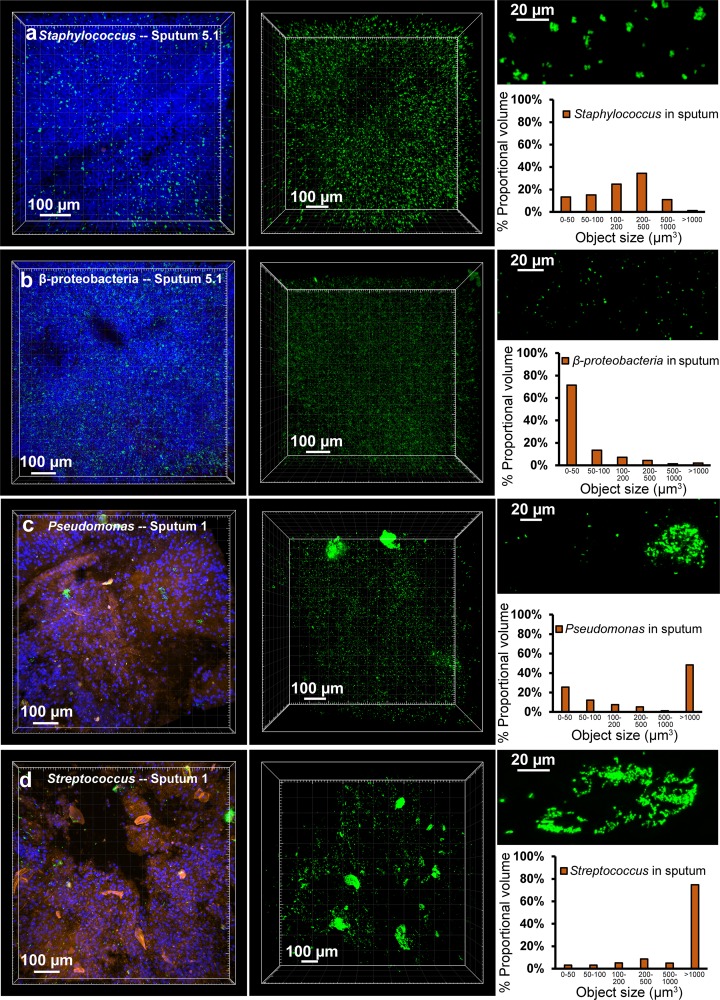
Aggregation patterns vary between species. (a) HCR with a *Staphylococcus*-specific probe in sputum sample 5.1 (green). The first panel is a maximum intensity projection of a *Z*-stack after HCR and staining with DAPI (blue) and WGA (orange), acquired with a 10× objective. The second panel is a maximum intensity projection of a separate *Z*-stack acquired with a 10× objective while only collecting HCR signal (*Staphylococcus*-specific probe mix with B4 amplifier and AlexaFluor 488-conjugated B4 hairpins) (7,910 objects analyzed). Each object identified in the second panel’s *Z*-stack was binned according to proportional object volume (each object’s fluorescent volume relative to the total fluorescent volume for that *Z*-stack; shown in the graph on the right). The top right panel is a maximum intensity projection of a *Z*-stack acquired with a 25× objective, highlighting a representative region from the same sputum sample. (b to d) The same analysis was applied to sputum 5.1 using a Betaproteobacteria-specific probe with B4 amplifier and AlexaFluor 488-conjugated B4 hairpin (21,255 objects analyzed) (b), to sputum 1 with a *Pseudomonas*-specific probe mixture with B4 amplifier and AlexaFluor 647-conjugated B4 hairpins (9,520 objects analyzed) (c), or a *Streptococcus*-specific probe mixture with AlexaFluor 488-conjugated B4 hairpins (4,603 objects analyzed) (d).

Once optimized for retention and identification of bacteria in sputum, we utilized MiPACT-HCR to measure bacterial aggregation *in situ*. Bacterial aggregates are thought to contribute to the persistence of pathogen populations in chronic infections, including those in CF patients (1, 28–30), yet direct evidence for this is sparse (6, 19, 31). We examined distribution patterns of *Staphylococcus* sp. in sputum sample 5.1 (culture positive for *S. aureus* and *A. xylosoxidans*) by using a *Staphylococcus*-specific probe. Cultured bacteria were analyzed in parallel with magnification ×25 sputum surveys to calibrate our expectations for the signal size of single bacterial cells (see Fig. S7 in the supplemental material). The mean fluorescence volume of objects in stationary-phase cultures of *S. aureus* was 12.1 µm^3^. In sputum, *Staphylococcus* cells existed in a range of intermediate aggregates, with only 6% of objects being greater than 1,000 µm^3^ (see Fig. S7a). The *Staphylococcus* size distribution in sputum cleared for 5 or 14 days was similar, signifying that clearing preserves a range of bacterial aggregate sizes (see Fig. S2b in the supplemental material). Taking advantage of the large-scale surveying enabled by MiPACT, we next acquired ×10 magnification *Z*-stacks of sputum sample 5.1, analyzing thousands of objects in sputum volumes of ~0.1 to 0.3 mm^3^. Like the ×25 magnification surveys, *Z*-stacks at ×10 magnification revealed that *Staphylococcus* was chiefly visible as small to medium aggregates (85% of objects ranged in size from 50 to 1,000 µm^3^) (see Fig. 2a and S7a). In contrast, Betaproteobacteria showed very little aggregation; 71% of objects fell in the smallest bin (<50 µm^3^) (Fig. 2b; see also Fig. S7b in the supplemental material). Because *S. aureus* is the most common pathogen cultured from CF patients (21), we monitored aggregation in samples from three distinct areas of sputum 5 (5.1A, 5.1B, and 5.1C) (see Fig. S8a in the supplemental material). Also, three temporal samples from patient 5 (5.1, 5.2 [103 days after 5.1], and 5.3 [1 day after 5.2]) and a sample from patient 4, also culture positive for *S. aureus*, were analyzed. All samples demonstrated a similar pattern of small- to medium-sized aggregates (see Fig. S8b). Next, we took advantage of the straightforward multiplexing enabled by HCR (15) to concurrently probe Betaproteobacteria and *Staphylococcus* sp. in different regions of sputum 5.1 (see Fig. S9 in the supplemental material). Both were present in all areas of sputum 5.1 tested, but their relative abundance differed between regions (see Fig. S9).

The only organism for which sputum 1 was culture positive was *P. aeruginosa*, and surveying at ×10 and ×25 magnifications with a *Pseudomonas*-specific probe mixture revealed small objects (0 to 50 µm^3^) and large aggregates (>1,000 µm^3^) (Fig. 2c; see also Fig. S7c in the supplemental material), consistent with prior imaging of smears of CF sputum and thin sections of explanted CF patient lungs (6, 19). While surveying sputum sample 1 with the EUB338 probe, we unexpectedly found bacteria with a distinctive filamentous morphology. Patient 1 had previously produced a sputum sample that was culture positive for *Streptococcus anginosus*, and probing sputum 1 with a *Streptococcus*-specific probe mixture revealed a dense bacterial population (Fig. 2d). The largest proportion of *Streptococcus* signal volume (which ranged from ~10 to 300,000 µm^3^ at ×10 magnification) came from large (>1,000 µm^3^) aggregates (Fig. 2d). *Streptococcus* is often missed in routine clinical culturing, highlighting the gap that is often observed between culture-dependent and culture-independent techniques (32, 33).

**FIG 3:**
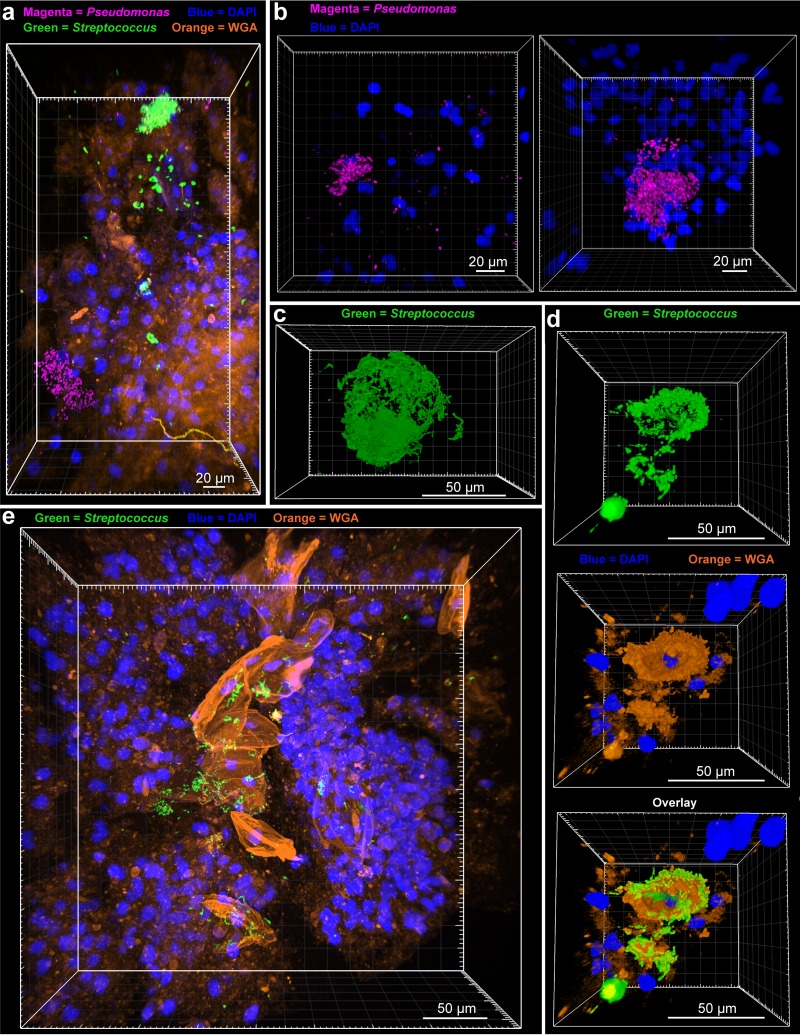
*Pseudomonas* and *Streptococcus* biofilm structure. (a) A maximum intensity projection was generated after HCR was performed on sputum 1 with a *Pseudomonas*-specific probe mixture (with B1 hairpins conjugated to AlexaFluor 647) and a *Streptococcus*-specific probe mixture (with B4 hairpins conjugated to AlexaFluor 488). (b) Maximum intensity projections showing *Pseudomonas* aggregates from sputum 1 after HCR with a *Pseudomonas* probe mixture and B4 hairpins conjugated to AlexaFluor 488 and DAPI staining. (c) Blend projection of a *Streptococcus* biofilm from sputum 1 (HCR with *Streptococcus* probe mixture with B4 hairpins conjugated to AlexaFluor 488). (d) Blend projections showing, stepwise, a *Streptococcus* aggregate (top; green), DAPI (blue), and WGA (orange) staining of host cells (middle), and an overlay of the two showing the arrangement of the *Streptococcus* biofilm around WGA-stained host cells (bottom). (e) Maximum intensity projection of HCR-identified *Streptococcus* (green), DAPI (blue), and WGA (orange) staining in sputum 1.

An important advantage of surveying large volumes at low magnification is the ability to quickly identify key areas that can benefit from higher magnification. After performing ×10 surveys in sputum, we focused on large bacterial aggregates with a 25× objective (Fig. 3). Multiplexing of sputum 1 for both *Streptococcus* and *Pseudomonas* revealed that aggregates were mostly monospecies, with little visible interaction (Fig. 3a). *Pseudomonas* aggregates existed in a range of sizes, with large biofilms having diameters up to ~50 µm (Fig. 3b). PMNs could be seen surrounding, and in some cases within, the biofilm structure (Fig. 3b). With finer resolution, it became apparent that the large *Streptococcus* aggregates visible at ×10 had morphologies indicative of association with an interior substrate (Fig. 3c and d). To determine the substrate, we stained samples of sputum 1 with DAPI and WGA after HCR with *Streptococcus*-specific probes. Staining revealed that the areas inside *Streptococcus* aggregates were in fact host cells with single-lobed nuclei (Fig. 3d). Each host cell boundary stained brightly with WGA, potentially indicative of polysaccharide moieties on the host cell surface (Fig. 3d and e). These results exemplify the ability of MiPACT-HCR to identify novel bacterium-host interactions.

**FIG 4:**
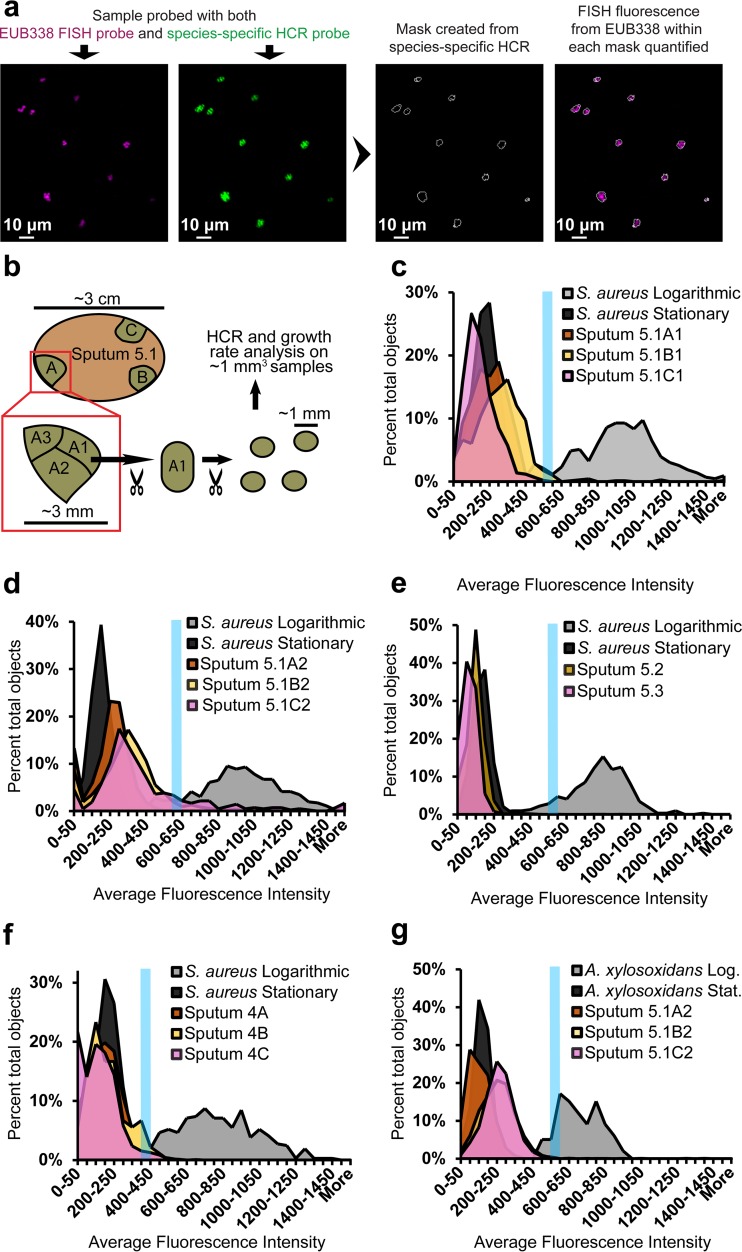
Growth rate estimates of CF pathogens *in situ.* (a) Diagram showing the process of estimating growth rates *in situ*. Samples were first stained with a species-specific B4 amplifier HCR probe, using B4 hairpins conjugated to AlexaFluor 488. Samples were then stained with the universal bacterial FISH probe EUB338, conjugated to two Cy5 fluorophores. Masks were made based upon HCR signal, and fluorescence intensity from FISH was quantified within each mask. (b) The basic sputum sampling technique. (c) For this and subsequent panels, *Z*-stacks of cultured cells and sputum samples were acquired with a 25× objective in parallel. The average fluorescence intensity of the FISH channel of each object is plotted on the *x* axis as a histogram. The blue line denotes the bin above which 90% of the logarithmic objects fell (for each particular experimental set). Growth rate analysis was performed on three distinct regions of sputum sample 5.1: 5.1A1 (409 objects analyzed), 5.1B1 (697 objects analyzed), and 5.1C1 (575 objects analyzed) (c), and on 5.1A2 (418 objects analyzed) 5.1B2 (1,087 objects analyzed), and 5.1C2 (419 objects analyzed) (d). (e) Analysis of temporal samples 5.2 (520 objects analyzed) and 5.3 (893 objects analyzed). (f) Analysis of three distinct regions of sputum 4: 4A (1,067 objects analyzed), 4B (73 objects analyzed), and 4C (599 objects analyzed). (g) Analysis of Betaproteobacteria from samples 5.1A2 (1,919 objects analyzed), 5.1B2 (2,351 objects analyzed), and 5.1C2 (1,523 objects analyzed).

While the importance of aggregative or biofilm modes of growth in chronic infection is well appreciated (1, 3, 4, 28–30, 34), the role of growth rate is less so. Recent studies demonstrated slow *in situ S. aureus* growth rates in CF sputum (35) and slow-growth-specific regulation networks in *P. aeruginosa* (36), underscoring the importance of careful growth measurements *in situ* for designing *in vitro* models that faithfully recapitulate *in vivo* physiology. Many species show a linear relationship between growth rate and rRNA abundance (37), but a number of challenges impede the calculation of precise growth rates in sputum from FISH data alone: rRNA abundance can be completely decoupled from growth rate in some species (37), at low growth rates rRNA abundance ceases to linearly correlate with growth rate (7), and sputum autofluorescence can overwhelm signals from slowly growing cells (see Fig. S4 in the supplemental material). To address these problems, we refrained from estimating specific growth rates of individual cells, instead opting to describe the growth rates of bacterial populations with respect to logarithmic- and stationary-phase standards, analyzed in parallel. We first verified that the FISH signal of both *S. aureus* and *A. xylosoxidans* decreased in stationary phase (see Fig. S10a in the supplemental material). We then determined that FISH signal from logarithmic cells did not substantially decay even after 14 days of clearing (see Fig. S10b). Lastly, we used HCR to distinguish bacterial signals from background autofluorescence and to select for the desired genus in a mixed population. For analysis, HCR-identified objects were outlined and EUB338 FISH fluorescence (the proxy for growth rate) within the outlines was quantified (Fig. 4a). EUB338 was chosen as the FISH probe due to its robustness and hybridization to a separate rRNA locus, preventing probe competition.

The growth rate measurements described above were performed on objects identified with a *Staphylococcus*-specific HCR probe from portions of sputum 5.1 taken from distinct areas of the sample (5.1A, -B, and -C, with subsamples 5.1A1, 5.1A2, etc.) (Fig. 4a and b). We then calculated the percentage of objects that crossed a threshold above which 90% of logarithmic-phase cultured cells fell (corresponding to a doubling time of ≈1 h). Regions 5.1A1, 5.1B1, and 5.1C1 demonstrated mostly low growth rates, with 0%, 4.3%, and 0.2% of objects reaching signal threshold (Fig. 4c). Interestingly, subsample 5.1C2 demonstrated an increase in growth rate compared to its neighbors, with 20.8%, of objects reaching threshold (no objects in 5.1A2 and 7.0% of objects in 5.1B2 reached threshold) (Fig. 4d). Temporal samples from patient 5 (sputum samples 5.2 and 5.3) did not reach threshold (Fig. 4e). Samples 4A and -B and -C, from a different *S. aureus* culture-positive patient, contained slowly growing bacteria as well, with only 2.7%, 4.1%, and 2.0% of objects, respectively, above threshold (Fig. 4f). In order to determine if aggregate size correlated with our proxy for growth rate, we separated *S. aureus* objects (from Fig. 4c to f) into quartiles with respect to object size and plotted against mean fluorescence intensity from FISH. Fluorescence intensity increased significantly with increasing object size, signifying that larger aggregates may have experienced higher growth rates (see Fig. S10c and d in the supplemental material). This was possibly due to the greater susceptibility of single cells to antibiotics (34). Interestingly, cultured, planktonic *S. aureus* cells also showed a positive correlation between object size and fluorescence intensity (see Fig. S10e). This is consistent with previous studies showing that RNA abundance and cell size increase with higher growth rates (38). Further study would be needed to determine what, if any, correlation exists between aggregate size and cell size *in situ*.

Lastly, as *A. xylosoxidans* is subject to the same *in vivo* conditions as *S. aureus* in these samples, we assayed growth rates of Betaproteobacteria in sputum sample 5.1. All populations were growing slowly, with 0.1% of objects from 5.1A2, 0.5% from 5.1B2, and 0.8% from 5.1C2 reaching the threshold set by logarithmic-phase *A. xylosoxidans* standards (doubling time of ≈0.6 h) (Fig. 4g).

We have shown that MiPACT-HCR is effective at retaining and visualizing bacteria in complex samples after optical clearing, and enables the rapid survey of large volumes of these samples. In our CF patient sputum samples, *P. aeruginosa*, *Streptococcus* sp., *A. xylosoxidans*, and *S. aureus* aggregation patterns varied, suggesting that broader, species-specific cellular interaction trends occur *in vivo*. Our results also reinforced that *in vivo* CF pathogen aggregates, particularly in regards to *S. aureus* and *A. xylosoxidans*, are considerably smaller than typical laboratory biofilms (34), an important observation when attempting to model biofilms for this context in the laboratory. We have also demonstrated that MiPACT-HCR, in combination with FISH, provides an accessible method for assessing growth rates *in situ*. Using this strategy, we found low growth rates for *S. aureus* and *A. xylosoxidans*, consistent with the few *in situ* measurements existing for these CF pathogens (6, 7, 35). While most of the populations surveyed were slow growers, there were pockets of relatively fast growth, illustrating the heterogeneity in *in vivo* growth rates that is beginning to be described in the literature (35). Indeed, due to concomitant expectoration of sputum plugs from different airways, and from the gradients of nutrients and oxygen existing with various sputum plug geometries (18), heterogeneity within the same expectorated sample is expected. Furthermore, particular sputum environments may favor one bacterial species or another, leading to the compositional heterogeneity that we observed in the same sputum sample.

MiPACT provides a widely accessible technique to characterize biogeography *in situ*, in three dimensions (3D) and at imaging depths not previously practical, and has the potential to reveal the range of microbes growing slowly in a wide variety of contexts. Through systematic application of MiPACT and fluorescent hybridization techniques such as HCR, patterns may emerge that will lead to new insights into heterogeneous polymicrobial communities, conditions conducive to promoting health (the human microbiome), treating disease (bacterial infections), or understanding important ecological interactions.

## MATERIALS AND METHODS

### Strains and growth conditions.

The following strains were used in this study: *Pseudomonas aeruginosa* PA14, *Staphylococcus aureus* MN8, and a clinical isolate of *Achromobacter xylosoxidans* generously donated by CHLA. Strains were grown aerobically in lysogeny broth (LB) at 37°C with shaking at 250 rpm.

### Growth curves studies and embedding bacteria in acrylamide-based hydrogel blocks.

Five-milliliter cultures of *S. aureus*, *P. aeruginosa*, and *A. xylosoxidans* were inoculated with single colonies from LB agar plates. Once cultures reached late exponential phase, the cultures were diluted 1:1,000 into glass culture tubes with 10 ml of medium. The optical density at 500 nm (OD_500_) or the OD_600_ of these cultures was tracked using a Thermo Spectronic 20D+ system during aerobic growth, with shaking, at 37°C. At intervals denoted in Fig. S10 in the supplemental material, cells were removed from the culture, normalized to an OD_500_ or OD_600_ of 1 in LB, and then PFA (to 2% [vol/vol]; EMS 15713) was added. Samples were slowly rotated at 4°C overnight. The next day, after washing with phosphate-buffered saline (PBS), fixed cells were diluted 1:10 into 4% (vol/vol) 29:1 acrylamide:bis-acrylamide (catalog number 161-0146; Bio-Rad) and 0.25% (wt/vol) VA-044 hardener (catalog number 27776-21-2l; Wako) in 1× PBS for polymerization. After leaving samples open, but covered, in an anaerobic hood for 5 min to decrease oxygen in the headspace, blocks were polymerized in a 37°C water bath for 3 h, without shaking, and then cut to ~1 mm^3^. Unless otherwise noted, blocks were cleared for 5 days in 8% SDS and then processed for FISH as described below. All solutions were sterilized with a 0.2-µm filter.

### Sputum sample collection.

Sputum samples were collected at CHLA in accordance with study CCI-13000211, which was approved by the CHLA IRB. Immediately upon expectoration, sputum samples were placed into 50-ml conical tubes with 25 ml of 4% paraformaldehyde solution in 1× PBS (pH 7.2). Samples were incubated for 24 h at 4°C, gently washed 3 times in 50 ml 1× PBS (pH 7.2), and then stored in 1× PBS with 0.01% (wt/vol) sodium azide at 4°C.

### MiPACT processing of sputum.

For sputum samples, small sections roughly 5 mm in diameter were removed under sterile conditions with a scalpel and placed in a 1.5-ml culture tube. Samples were incubated overnight in 4% (vol/vol) 29:1 acrylamide:bis-acrylamide and 0.25% (wt/vol) VA-044 hardener in 1× PBS, made fresh and filter sterilized. After overnight incubation, samples were moved into an anaerobic hood and left open, but covered, for 5 min to remove headspace oxygen. Samples were polymerized in fresh solution for 3 h at 37°C in a water bath, without shaking. Under sterile conditions, samples were routed to a solution of 8% SDS, pH 8.0, at 37°C, with shaking until cleared. Generally, samples took from 3 to 14 days to fully clear (average, 5 days). After clearing, samples were washed 3 times in 50-ml conical tubes in 1× PBS (ml volumes) to remove SDS. Once cleared, samples were stored in 1× PBS with 0.01% (wt/vol) sodium azide and 1× ProtectRNA RNase inhibitor (catalog number R7397; Sigma) at 4°C. PACT has a flexible formulation with application-specific recommendations regarding inclusion or exclusion of PFA and bis-acrylamide (see the troubleshooting instructions of Treweek et al. in reference 12).

### Lysozyme and lysostaphin digestion.

Before lysozyme/lysostaphin treatment, samples were trimmed and sectioned with ethanol-sterilized razor blades to ~1-mm^3^ blocks. All sputum and cultured cells in acrylamide-based hydrogel blocks were incubated in 1.5-ml microcentrifuge tubes in 500 µl of a sterile solution of lysozyme (1 mh/ml; catalog number L6876; Sigma) and lysostaphin (0.05 mg/ml; catalog number L7386; Sigma) in 10 mM Tris-HCl (pH 7.6) for 3 h at 37°C with shaking. Samples were then washed 2 times for 30 min each in 50 ml of 1× PBS.

### FISH.

To ensure adequate binding site saturation of our target rRNAs, we evaluated using FISH over a range of probe concentrations, with loss of binding site saturation occurring at around 0.008 ng/µl (1.2 nM) (see Fig. S4 in the supplemental material). To accommodate potential sample variability, we chose a higher concentration, 1 µg/ml (150.7 nM), for subsequent FISH experiments. Therefore, unless otherwise noted, samples were hybridized with 1 µg/ml (150.7 nM) probe at 46°C, with shaking, overnight in 15% formamide for cultured cells in acrylamide-based hydrogel blocks or 25% formamide for sputum samples (or growth rate standards). Each ~1-mm^3^ sample was incubated in 500 µl hybridization buffer (180 µl of 5 M NaCl, 20 µl of 1 M Tris-HCl [pH 7.6], 2 µl 5% [wt/vol] SDS, 150 or 250 µl formamide [for 15% and 25% formamide solutions, respectively], and Milli-Q H_2_O to 1 ml) in a 1.5-ml culture tube. All solutions were filter sterilized (0.2-µm filter). To remove excess probe, cultured cells in hydrogel blocks were washed in 50 ml 337.5 mM FISH wash buffer (3,375 µl of 5 M NaCl, 1 ml of Tris-HCl [pH 7.6], 500 µl of 0.5 M EDTA [pH 7.2], 100 µl of 5% SDS, and Milli-Q H_2_O to 50 ml) at 48°C for 6 h in a water bath, without shaking. Embedded sputum samples, and also cultured cells in hydrogel blocks used as standards for sputum experiments, were washed in 84 mM FISH wash buffer (840 µl of 5 M NaCl, 1 ml of Tris-HCl [pH 7.6], 500 µl of 0.5 M EDTA [pH 7.2], 100 µl of 5% SDS, and Milli-Q H_2_O to 50 ml) at 48°C for 6 h in a water bath, without shaking. Samples were then incubated in 250 µl refractive index matching solution (RIMS) (12) (40 g of HistoDenz; catalog number D2158; Sigma) in 30 ml of 0.02 M phosphate buffer with 0.1% Tween 20 and 0.01% sodium azide) with 10 µg/ml DAPI at room temperature (RT) with gentle shaking, protected from light, for at least 24 h before imaging.

### HCR.

HCR involves a hybridization step with an unlabeled DNA probe. This probe contains a specific sequence tag (the specific sequences used here were termed B1, B3, and B4) that triggers the oligomerization of pairs of fluorescently labeled DNA hairpins (the amplification step; for more details, see Choi et al. [15]).

### (i) Hybridization.

Samples were hybridized in 500 µl of HCR hybridization buffer (100 µl of 20× sodium chloride-sodium citrate [SSC], 100 mg dextran sulfate [catalog number D6001; Sigma], 200 µl formamide [for a 20% formamide solution] or 250 µl formamide [for a 25% formamide solution], and Milli-Q H_2_O to 1 ml) with 30 nM initiator probe at 46°C, with shaking, for 24 or 48 h. For EUB338, NON338, and STA3 probes, 25% formamide buffer was used. For the *Streptococcus* probe mixture (Str and Str56 probes, each at 20 nM), the *Pseudomonas* probe mixture (PseaerA, PseaerB, Pae997, and PSE227; each at 20 nM), and for BET42a, 20% formamide was used. All solutions were filter sterilized. Excess probe was removed by washing each sample in 50 ml of 42 mM FISH wash buffer (420 µl of 5 M NaCl, 1 ml of Tris-HCl [pH 7.6], 500 µl of 0.5 M EDTA [pH 7.2], 100 µl of 5% SDS, and Milli-Q H_2_O to 50 ml) at 52°C for 6 h in a water bath, without shaking. For sputum samples from patients 4 and 5, samples were hybridized for 48 h. For sputum samples from patient 1, samples were hybridized for 24 h.

### (ii) Amplification.

Before amplification, hairpin pairs were heated to 95°C for 1.5 min in a thermocycler in separate PCR tubes. Hairpins were then cooled at room temperature (RT) for at least 30 min while protected from light. Each hairpin in a pair was added at 1:25 from a 3 µM stock to a final concentration of 120 nM in HCR amplification buffer (100 µl 20× SSC, 100 mg dextran sulfate, and Milli-Q H_2_O to 1 ml) for acrylamide-based hydrogel blocks and sputum 1, or at 1:12.5 to a final concentration of 240 nM for sputum from patients 5 and 4. A 120-μl volume of amplification buffer with the appropriate hairpin mixture was then added to each sample in a 1.5-ml centrifuge tube. Samples were incubated at RT with gentle shaking for 48 h. For sputum samples from patients 4 and 5, samples were amplified for 48 h. For sputum samples from patient 1, samples were amplified for 24 h. After amplification, samples were washed in 50 ml of 337.5 mM FISH wash buffer at 48°C for 3 h in a water bath, without shaking. Samples were then incubated in 250 µl RIMS with 10 µg/ml DAPI (1:1,000 from 10-mg/ml stock solutions in dimethyl sulfoxide) at RT with gentle shaking for at least 24 h before imaging.

FISH probes were dilabeled with the indicated fluorophores, with one fluorophore at the 5′ end and one at the 3′ end. Three HCR initiator/hairpin systems were used in this study: B1, B3, and B4. For B1 initiator probes, the sequence 5′-TATAGCATTCTTTCTTGAGGAGGGCAGCAAACGGGAAGAG-3′ was added to the 3′ end of the indicated DNA probe. For B3 initiator probes, 5′-TAAAAAAGTCTAATCCGTCCCTGCCTCTATATCTCCACTC-3′ was added to the 3′ end of the indicated DNA probe.

For B4 initiator probes, 5′-ATTTCACATTTACAGACCTCAACCTACCTCCAACTCTCAC-3′ was added to the 3′ end of the indicated DNA probes. DNA hairpins conjugated to either AlexaFluor 488, AlexaFluor 594, or AlexaFluor 647, as indicated, were used with the appropriate initiator probe sets. Hairpins conjugated to fluorophores were purchased from Molecular Instruments.

### Lectin staining.

When indicated, lectin staining was performed immediately before incubation in RIMS/DAPI. WGA conjugated to rhodamine (vector RL-1022) was used for lectin staining. Samples were incubated in 1 ml of 50 µg/ml WGA in 1× PBS at RT, with shaking, for 24 h. They were then washed for another 24 h at RT, with shaking, in 1 ml of 1× PBS before incubation in RIMS/DAPI.

### Imaging.

Prior to imaging, samples were incubated at RT overnight, with shaking, in RIMS with 1 µg/ml DAPI. Samples were then mounted on slides in 0.9 mm or 1.7 mm CoverWell perfusion chambers (Electron Microscopy Services) with a coverslip on the top. Imaging was performed using a Zeiss LSM 780 confocal microscope or a Zeiss LSM 880 confocal microscope with either a Plan-Apochromat 10×/0.45-numerical aperture M27 objective (working distance [wd], 2.0 mm) or an LD LCI Plan-Apochromat 25×/0.8-numerical aperture Imm Corr DIC M27 multi-immersion objective (wd, 0.57 mm), using glycerol as the immersion fluid. All images and *Z*-stacks were collected in 12-bit mode, with at least a 1,024-by-1,024 scan format and a line averaging of 2. Image reconstructions were made with Imaris imaging software (Bitplane) or the FIJI distribution of ImageJ (39, 40). Image analysis was chiefly performed using the 3D object counter plug-in (41) in FIJI to identify and quantify fluorescently labeled objects. The R package was used to make box plots and for *t* test analysis (42).

### Aggregation analysis.

Aggregation measurements were performed from HCR-stained samples using probes listed in Table 1 (probes that did not withstand the HCR wash step are also shown). Laser power and gain settings were adjusted for each sample so that the brightest objects were just below saturation. For image analysis, the 3D object counter from ImageJ was utilized to record the fluorescence volume of each object. Objects were then binned according to volume. The fluorescence volume of each object in a given bin was summed, and each bin sum was divided by the total fluorescent volume of the entire *Z*-stack to obtain a proportional volume value for each bin.

**TABLE 1:** DNA probes used in this study

Probe (reference), description	Sequence (5′–3′)
Probes used successfully	
EUB338 (43), universal	GCTGCCTCCCGTAGGAGT
NON338 (44), reverse complement of EUB338	ACTCCTACGGGAGGCAGC
PseaerA (45), for *Pseudomonas*	GGTAACCGTCCCCCTTGC
PseaerB (45), for Pseudomonas	TCTCGGCCTTGAAACCCC
Pae997 (46), for Pseudomonas	TCTGGAAAGTTCTCAGCA
PSE227 (47), for *Pseudomonas*	AATCCGACCTAGGCTCATC
Str (48), for *Streptococcus*	CACTCTCCCCTTCTGCAC
Str56 (49), for *Streptococcus*	ATCCTGCGTTCTACTTGC
BET42a (50), for Betaproteobacteria	GCCTTCCCACTTCGTTT
STA3 (51), for *Staphylococcus*	GCACATCAGCGTCAGT
Universal 515 (52)	CGTATTACCGCGGCTGCTGGCAC
Probes that did not withstand HCR wash conditions	
Ppu646 (53), for *Pseudomonas*	CTACCGTACTCTAGCTTG
Staaur-16S69 (54), for *Staphylococcus*	GAAGCAAGCTTCTCGTCCG
Ach-221 (55), for *A. xylosoxidans*	CGCTCYAATAGTGCAAGGTC

### Growth rate analysis.

For growth rate measurements, HCR was performed on sputum samples and, in parallel, on logarithmic or stationary-phase bacteria (of the appropriate species) embedded in acrylamide-based hydrogel blocks that had been cleared for 5 days (unless otherwise noted). All HCR for growth rate measurements was performed with hairpins conjugated to Alexafluor488. After the typical HCR hairpin wash, all samples were hybridized with EUB338 di-labeled with CY5 in 25% formamide for 24 h. Samples were then washed in 84 mM FISH wash buffer for 3 h at 48°C in a water bath with no shaking. Samples were incubated with RIMS/DAPI for at least 24 h at RT with gentle shaking before imaging. During image acquisition, laser power and gain settings were adjusted for each sample in the HCR channel (Alexafluor488) so that the brightest objects were just below saturation. Laser power and gain settings for the FISH channel (CY5) were adjusted for so that the brightest objects in logarithmic phase culture standards were just below saturation. Once adjusted for logarithmic standards, the FISH settings were kept constant for the stationary-phase standard and for all sputum samples. For image analysis, the 3D object counter from ImageJ was used to perform segmentation from the HCR channel. The redirect option was used to measure fluorescence of each HCR-identified object in the FISH channel. The threshold and minimum size settings in 3D object counter were kept constant for all samples in a set. Objects were binned by average fluorescence intensity, and then the relative frequency of each bin was calculated by dividing the number of objects in each bin by the total number of objects for each sample. Histograms were then created from the relative frequency of each bin.

### Colocalization analysis.

For colocalization analysis, HCR was performed on sputum samples with two separate probes and two corresponding hairpin sets, one conjugated to AlexaFluor488 and one conjugated to Alexafluor647. *Z*-stacks were obtained with a 25× objective. The 3D object counter plug-in was used for each channel to obtain threshold images and identify objects with a minimum size of 5 voxels. The two binary *Z*-stacks (one from each channel) were multiplied together, and objects in the product *Z*-stack (with a size of at least 3 voxels) were counted with a 3D object counter. The number of objects in the product *Z*-stack was divided by the number of objects in each original *Z*-stack to yield the percent colocalization.

## SUPPLEMENTAL MATERIAL

Figure S1  Intrasample sputum composition. (A) Diagram demonstrating how sputum samples were processed for confocal microscopy analysis. (B) Blend projections of three regions from sputum 1 (top row) and sputum 5.1 (bottom row) after staining with DAPI (blue) and WGA (orange), from *Z*-stacks acquired with a 10× objective. Download Figure S1, JPG file, 2.6 MB

Figure S2  Bacterial retention after clearing. (A) Fixed, embedded *S. aureus* and *P. aeruginosa* cultured cells harvested in logarithmic or stationary phase were stored in 1× PBS at 4°C for 14 days (cleared at 0 days) or cleared in 8% SDS in 1× PBS at 37°C for 14 days. Samples were then incubated in RIMS with DAPI. A *Z*-stack was collected (400 to 1,500 cells/*Z*-stack), and the number of DAPI-stained cells per ml was calculated. Each bar represents the result from technical triplicates, and error bars represent standard errors. (B) The same region of sputum 5.1 was cleared for either 5 or 14 days. HCR with STA3-B4 and hairpins conjugated to AlexaFluor 488 was then performed, and *Z*-stacks with a 25× objective were acquired from each sample. A total of 856 objects from the sample cleared for 5 days and 928 objects from the sample cleared for 14 days were analyzed. The percent proportional volume was calculated for each sample. Download Figure S2, JPG file, 0.4 MB

Figure S3  Lysozyme/lysostaphin requirement for FISH of *S. aureus* cells. (A) FISH was performed with a dilabeled EUB338 probe (labeled with Cy3) at various concentrations on logarithmic-phase *P. aeruginosa* cells in bis-acrylamide-based hydrogel blocks after clearing for 5 days. *Z*-stacks were collected with a 25× objective, and the integrated density (sum of all pixel intensity values for a given object) per cell was calculated using the 3D object counter plug-in from ImageJ. Error bars represent standard errors, with sample sizes for probe concentrations ranging from 0.24 nM to 753.25 nM of 430, 414, 490, 489, 395, and 367 cells, respectively. (B) After clearing for 5 days, logarithmic-phase *P. aeruginosa* or *S. aureus* cells were washed in 1× PBS, and half the samples were digested with lysozyme/lysostaphin. FISH was then performed with a dilabeled EUB338 probe (labeled with AlexaFluor 594), and samples were stained with DAPI before imaging in RIMS. *Z*-stacks with a 25× objective were collected with the same laser power/gain settings, and maximum intensity projections are shown. Download Figure S3, JPG file, 1 MB

Figure S4  Autofluorescence in sputum. FISH using the dilabeled EUB338 FISH probe indicated or HCR using EUB338 was performed on stationary- or logarithmic-phase *P. aeruginosa* in bis-acrylamide-based hydrogel blocks after clearing. Average fluorescence per cell was calculated from single-plane images acquired with a 25× objective. The same laser/gain settings used to image bacteria were used to take images of cleared sputum sample 5.1. A histogram showing average fluorescence per cell (blue) is shown, with a histogram denoting pixel intensity values from sputum autofluorescence for the respective fluorophore acquisition settings (orange). Download Figure S4, JPG file, 1.2 MB

Figure S5  Cross-reactivity of HCR probes. HCR was performed on sputum samples 1 and 5.1 with the indicated probes/probe mixes and stained with DAPI. The panels depict 10-plane maximum intensity projections acquired with a 10× objective. EUB338-B4 was used with hairpin conjugated to AlexaFluor 647. NON338-B4 was used with hairpin conjugated to AlexaFluor 647. The *Pseudomonas* mix-B4 was used with hairpin conjugated to AlexaFluor 594. The *Streptococcus* probe mix-B4 was used with hairpin conjugated to AlexaFluor 594. STA3-B3 was used with hairpin conjugated to AlexaFluor 488. BET42a-B4 was used with hairpin conjugated to AlexaFluor 594. All bacteria are shown in green, and the results of DAPI staining (blue) are included to show sputum architecture. Laser power/gain settings were kept constant for both images acquired with a particular probe set (for NON338, the same laser power/gain settings as those for EUB338 were used). Download Figure S5, JPG file, 2.3 MB

Figure S6  Object-based colocalization in sputum with HCR. Multiplex HCR was performed on sputum 1 or sputum 5.1 with two concurrent initiator probes (denoted in the figure). Both Str and Str56 were used for *Streptococcus* (*Streptococcus* mix), and PseaerA, PseaerB, Pae997, and PSE227 were used for *Pseudomonas* (Pseudomonas mix). Amplification was performed with one initiator probe set conjugated to AlexaFluor 488 (green) and one conjugated to AlexaFluor 647 (magenta). *Z*-stacks were acquired with a 25× objective, and maximum intensity projections are shown. Object-based colocalization was performed by calculating the percentage of objects in each channel that overlapped with objects in the other channel. Values are listed in table in the upper right panel. Download Figure S6, JPG file, 2 MB

Figure S7  Size distribution of sputum aggregates compared to cultured cells. HCR was performed on cultured stationary-phase cells after fixing and embedding in acrylamide-based hydrogel blocks. *Z*-stacks were acquired with a 25× objective, and objects were binned according to proportional size (relative to the total fluorescent HCR volume of a sample). (A) Cultured stationary-phase *S. aureus* cells were compared to objects identified in sputum 5.1 with the *Staphylococcus*-probe mixture B4 with hairpins conjugated to AlexaFluor 647 (1,223 sputum objects analyzed). The *Staphylococcus* in sputum 5.1 image shows one panel out of four that were imaged and analyzed to obtain the histogram. (B) Cultured *A. xylosoxidans* harvested at stationary phase was compared to objects identified in sputum 5.1 with BET42a-B4 and hairpins conjugated to AlexaFluor 488 (4,091 sputum objects analyzed). (C) Cultured, stationary-phase *P. aeruginosa* was compared to objects identified in sputum 1 with a *Pseudomonas*-specific probe mixture and hairpins conjugated to AlexaFluor 647 (1,110 sputum objects analyzed). Download Figure S7, JPG file, 1.6 MB

Figure S8  Intersample and interpatient *S. aureus* aggregation patterns (A) Diagram demonstrating how sputum samples were processed for confocal microscopy analysis. (B) Three different sections of sputum 5.1 were probed with *Staphylococcus*-specific probe STA3 with a B4 amplifier and B4 hairpins conjugated to AlexaFluor 647: 5.1A1 (2,852 objects analyzed), 5.1B1 (2,892 objects analyzed), and 5.1C1 (1,992 objects analyzed). Maximum intensity projections are shown. As in Fig. 2, objects were binned according to proportional size and histograms were graphed. The same analysis was performed on three samples collected at three different time points from patient 5, (STA3 with B4 amplifier and AlexaFluor 647 conjugated B4 hairpins): 5.1 (4,250 objects analyzed), 5.2 (103 days after 5.1; 3,714 objects analyzed), and 5.3 (1 day after 5.2; 2,574 objects analyzed), and on three separate sections of a sputum sample from patient 4 (STA3 with B4 amplifier and AlexaFluor 594-conjugated B4 hairpins): 4A (825 objects analyzed), 4B (4,538 objects analyzed), and 4C (3,043 objects analyzed). All *Z*-stacks were acquired with a 10× objective. Download Figure S8, JPG file, 2.4 MB

Figure S9  HCR multiplexing shows heterogeneity in the ratios of Betaproteobacteria to *Staphylococcus* in sputum sample 5.1. HCR with BET42a-B1 and hairpins conjugated to AlexaFluor 647 and STA3-B4 and hairpins conjugated to AlexaFluor 488 was performed on three separate regions of sputum sample 5.1. *Z*-stacks at 10× were acquired, and maximum intensity projections were produced (top row). To determine relative abundances of *Staphylococcus* and Betaproteobacteria in each section of sputum 5.1, the total fluorescence volume from the BET42a signal was divided by the total fluorescence volume from the STA3 signal. After DAPI staining, 25× *Z*-stacks were acquired, and blend projections were produced (bottom row). Download Figure S9, JPG file, 2.6 MB

Figure S10  Growth rate controls and analysis. (A) Optical density of *S. aureus* and *A. xylosoxidans* cultures for growth curves plotted with the integrated density of cell fluorescence obtained by FISH with a EUB338 probe dilabeled with Cy3 from culture samples taken at each point along the growth curve. Error bars represent standard errors. For *A. xylosoxidans*, sample sizes from the earliest time point (5.5 h) to the last time point (30.5 h) were 78, 150, 465, 1,276, 1,504, and 1,303 cells, respectively. For *S. aureus*, sample sizes from the earliest time point (4 h) to the last time point (29 h) were 318, 954, 795, 1,139, 2,738, 395, and 193 cells, respectively. (B) Average fluorescence intensity shown for *S. aureus* logarithmic cultures cleared for 5 days (used as the standard in growth rate experiments) and 14 days, compared to *S. aureus* stationary-phase cultures cleared for 5 days (used as the standard in growth rate experiments). Growth rate measurements were performed as described for Fig. 4, with a STA3 HCR probe and hairpins conjugated to AlexaFluor 488, and a EUB338 FISH probe conjugated to Cy5. (C) All 6,757 STA3-positive objects that were analyzed from sputum samples 4, 5.1, 5.2, and 5.3 (Fig. 4) were broken up into quartiles by object size and graphed on a scatterplot of object size versus average fluorescence intensity. Box plots show average fluorescence intensity of the four quartiles for sputum samples (D) and for stationary-phase cultured *S. aureus* cells (E). A Welch’s two sample *t* test was applied to each adjacent quartile pair for both plots. Quartile 2 had a significantly increased average fluorescent intensity compared to quartile 1 (*P* < 2.2e−16 [D]; *P* = 1.2e−5 [E]), quartile 3 had a significantly increased average fluorescent intensity compared to quartile 2 (*P* < 1.2e−13 [D], *P* < 2.2e−16 [E]), and quartile 4 had a significantly increased average fluorescent intensity compared to quartile 3 (*P* < 2.2e−16 [D]; *P* < 2.2e−16 [E]). Plotting and analyses for the results shown in panels C, D, and E were performed in R. Download Figure S10, JPG file, 0.8 MB
