# Supplementary figures and images for: Exposing the Three-Dimensional Biogeography and Metabolic States of Pathogens in Cystic Fibrosis Sputum via Hydrogel Embedding, Clearing, and rRNA Labeling
Source: mBio. 2016 Sep 27;7(5):e00796-16. doi: 10.1128/mBio.00796-16 (PMC5040109; doi:10.1128/mBio.00796-16)

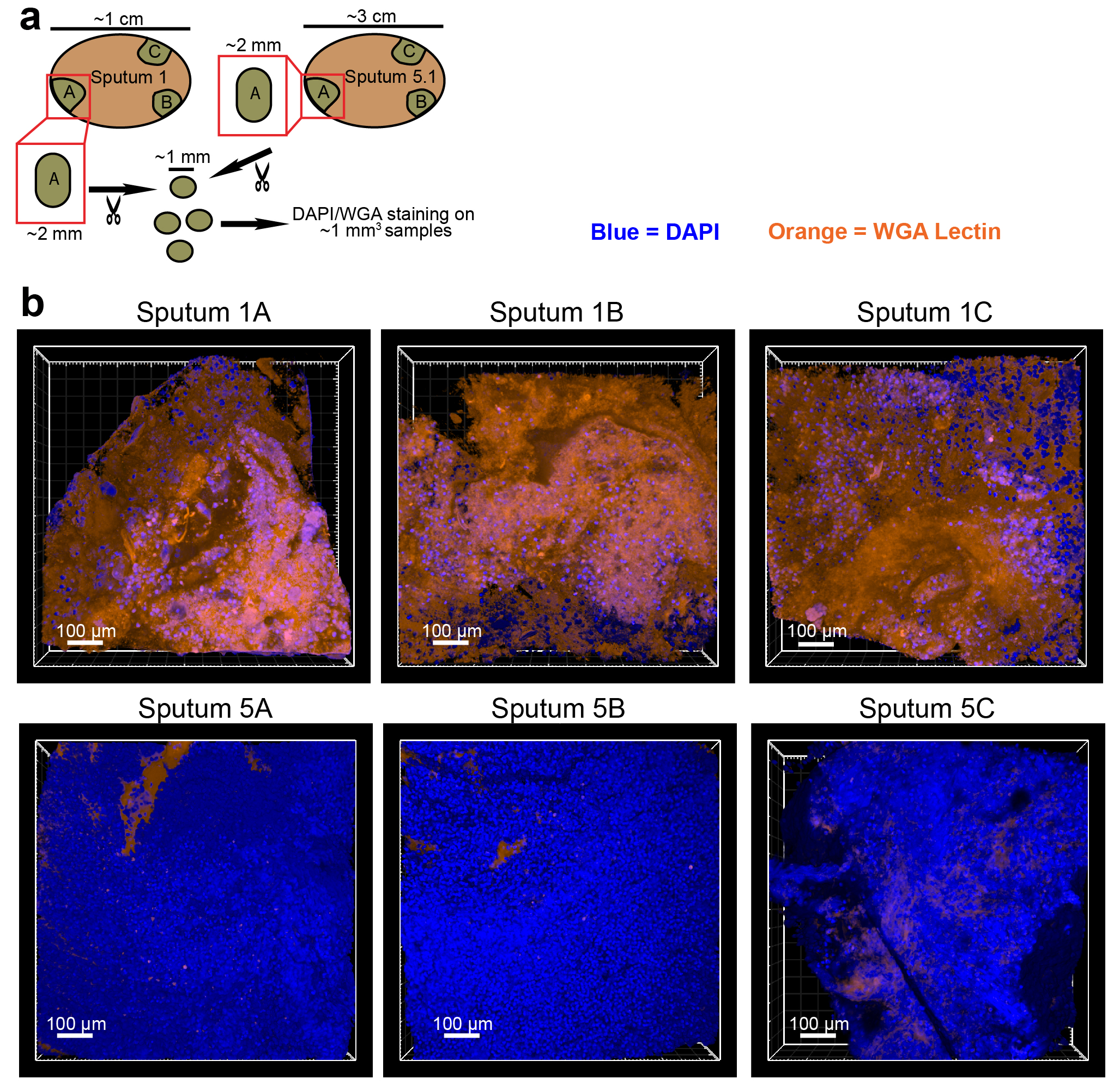

Supplement: Figure S1 — Intrasample sputum composition. (A) Diagram demonstrating how sputum samples were processed for confocal microscopy analysis. (B) Blend projections of three regions from sputum 1 (top row) and sputum 5.1 (bottom row) after staining with DAPI (blue) and WGA (orange), from Z-stacks acquired with a 10× objective. Download [file mbo004162984sf1.jpg]

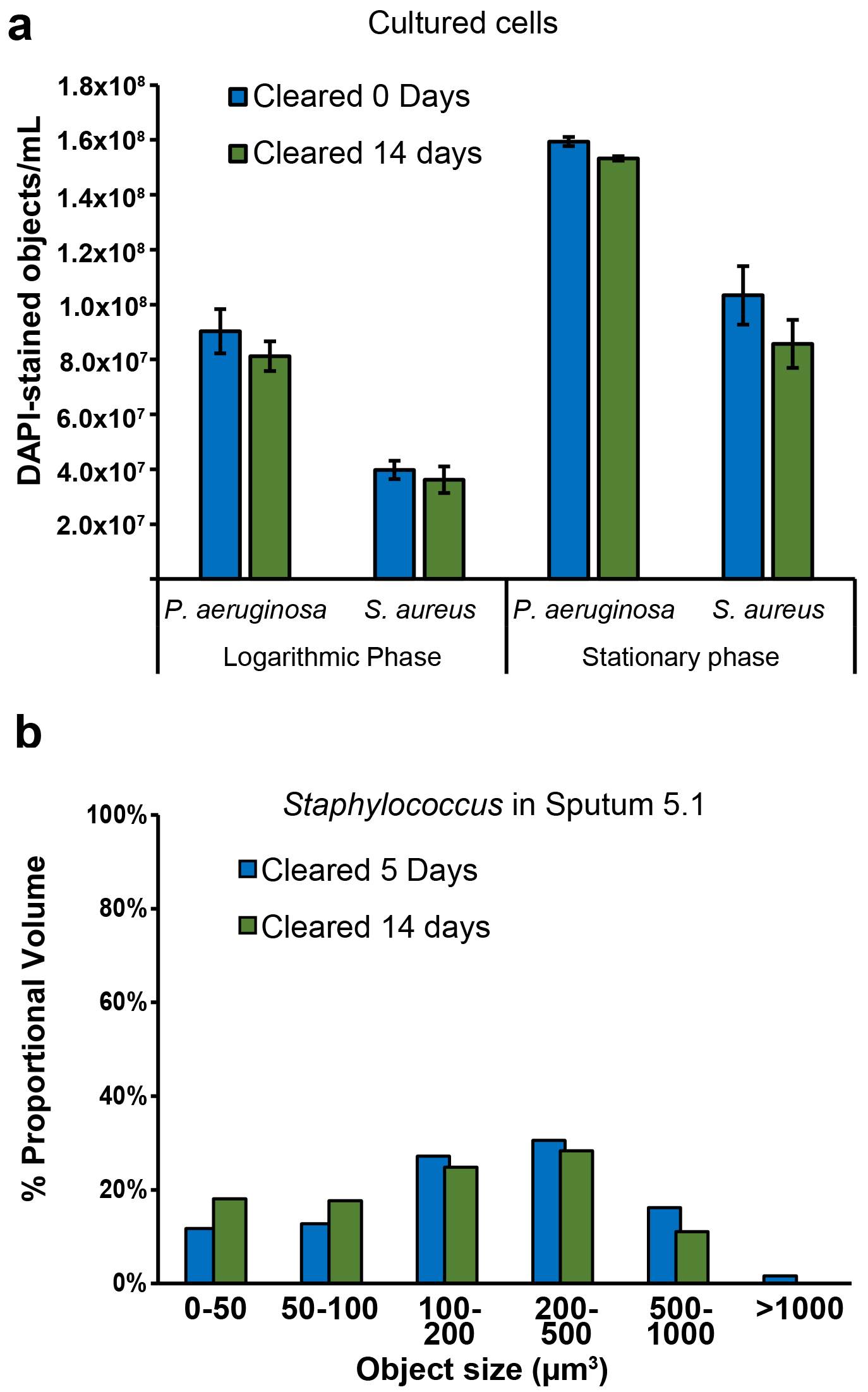

Supplement: Figure S2 — Bacterial retention after clearing. (A) Fixed, embedded S. aureus and P. aeruginosa cultured cells harvested in logarithmic or stationary phase were stored in 1× PBS at 4°C for 14 days (cleared at 0 days) or cleared in 8% SDS in 1× PBS at 37°C for 14 days. Samples were then incubated in RIMS with DAPI. A Z-stack was collected (400 to 1,500 cells/Z-stack), and the number of DAPI-stained cells per ml was calculated. Each bar represents the result from technical triplicates, and error bars represent standard errors. (B) The same region of sputum 5.1 was cleared for either 5 or 14 days. HCR with STA3-B4 and hairpins conjugated to AlexaFluor 488 was then performed, and Z-stacks with a 25× objective were acquired from each sample. A total of 856 objects from the sample cleared for 5 days and 928 objects from the sample cleared for 14 days were analyzed. The percent proportional volume was calculated for each sample. Download [file mbo004162984sf2.jpg]

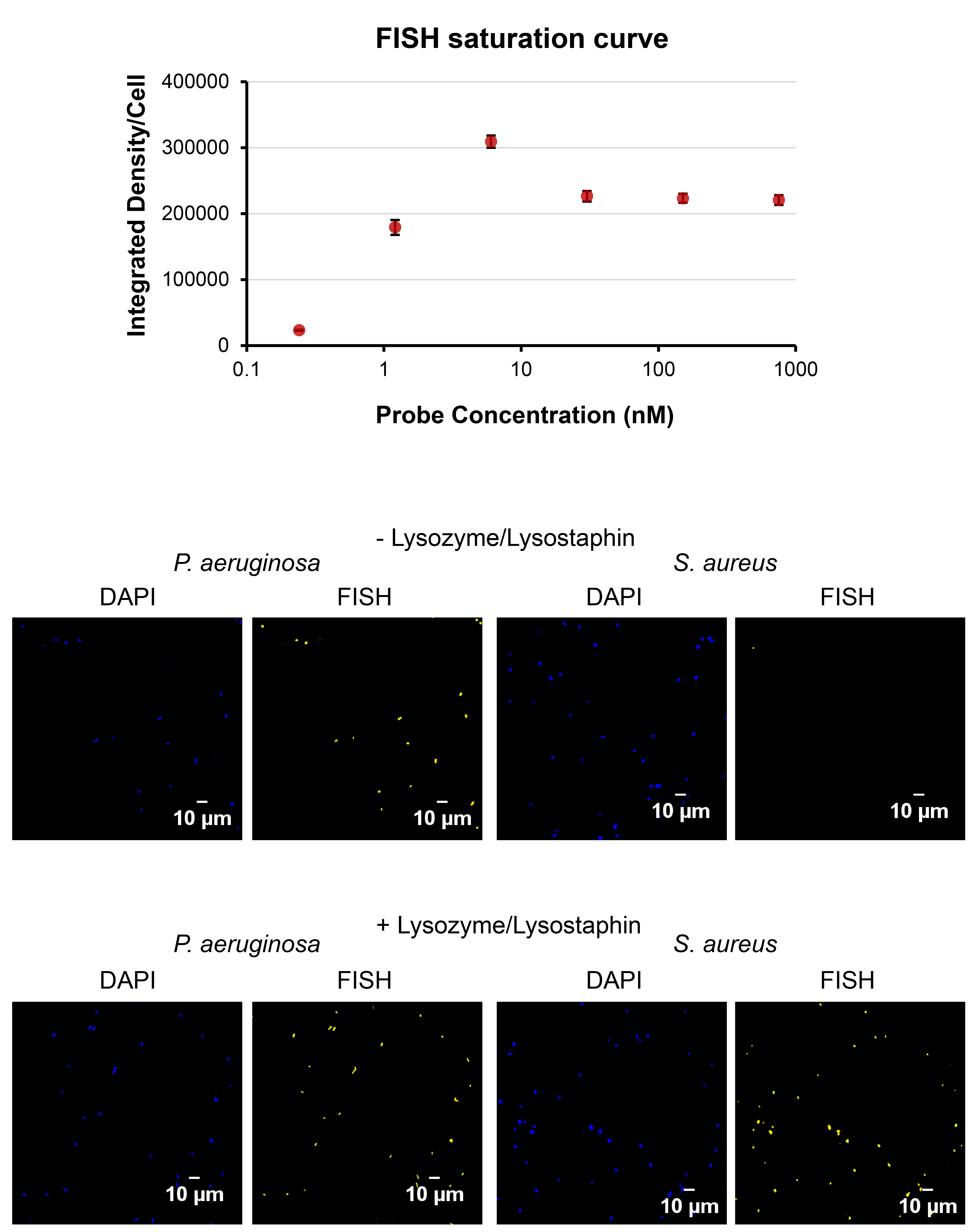

Supplement: Figure S3 — Lysozyme/lysostaphin requirement for FISH of S. aureus cells. (A) FISH was performed with a dilabeled EUB338 probe (labeled with Cy3) at various concentrations on logarithmic-phase P. aeruginosa cells in bis-acrylamide-based hydrogel blocks after clearing for 5 days. Z-stacks were collected with a 25× objective, and the integrated density (sum of all pixel intensity values for a given object) per cell was calculated using the 3D object counter plug-in from ImageJ. Error bars represent standard errors, with sample sizes for probe concentrations ranging from 0.24 nM to 753.25 nM of 430, 414, 490, 489, 395, and 367 cells, respectively. (B) After clearing for 5 days, logarithmic-phase P. aeruginosa or S. aureus cells were washed in 1× PBS, and half the samples were digested with lysozyme/lysostaphin. FISH was then performed with a dilabeled EUB338 probe (labeled with AlexaFluor 594), and samples were stained with DAPI before imaging in RIMS. Z-stacks with a 25× objective were collected with the same laser power/gain settings, and maximum intensity projections are shown. Download [file mbo004162984sf3.jpg]

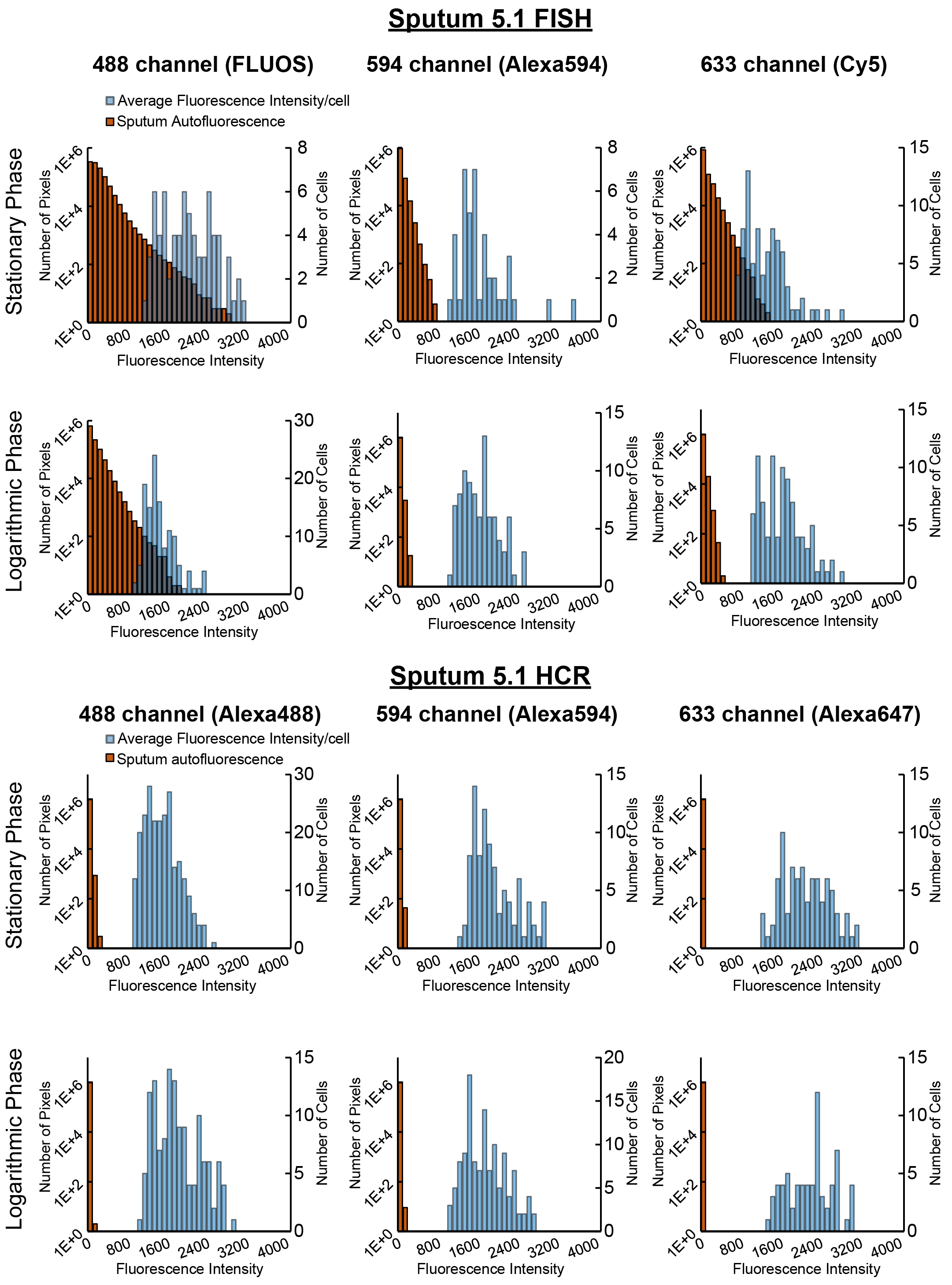

Supplement: Figure S4 — Autofluorescence in sputum. FISH using the dilabeled EUB338 FISH probe indicated or HCR using EUB338 was performed on stationary- or logarithmic-phase P. aeruginosa in bis-acrylamide-based hydrogel blocks after clearing. Average fluorescence per cell was calculated from single-plane images acquired with a 25× objective. The same laser/gain settings used to image bacteria were used to take images of cleared sputum sample 5.1. A histogram showing average fluorescence per cell (blue) is shown, with a histogram denoting pixel intensity values from sputum autofluorescence for the respective fluorophore acquisition settings (orange). Download [file mbo004162984sf4.jpg]

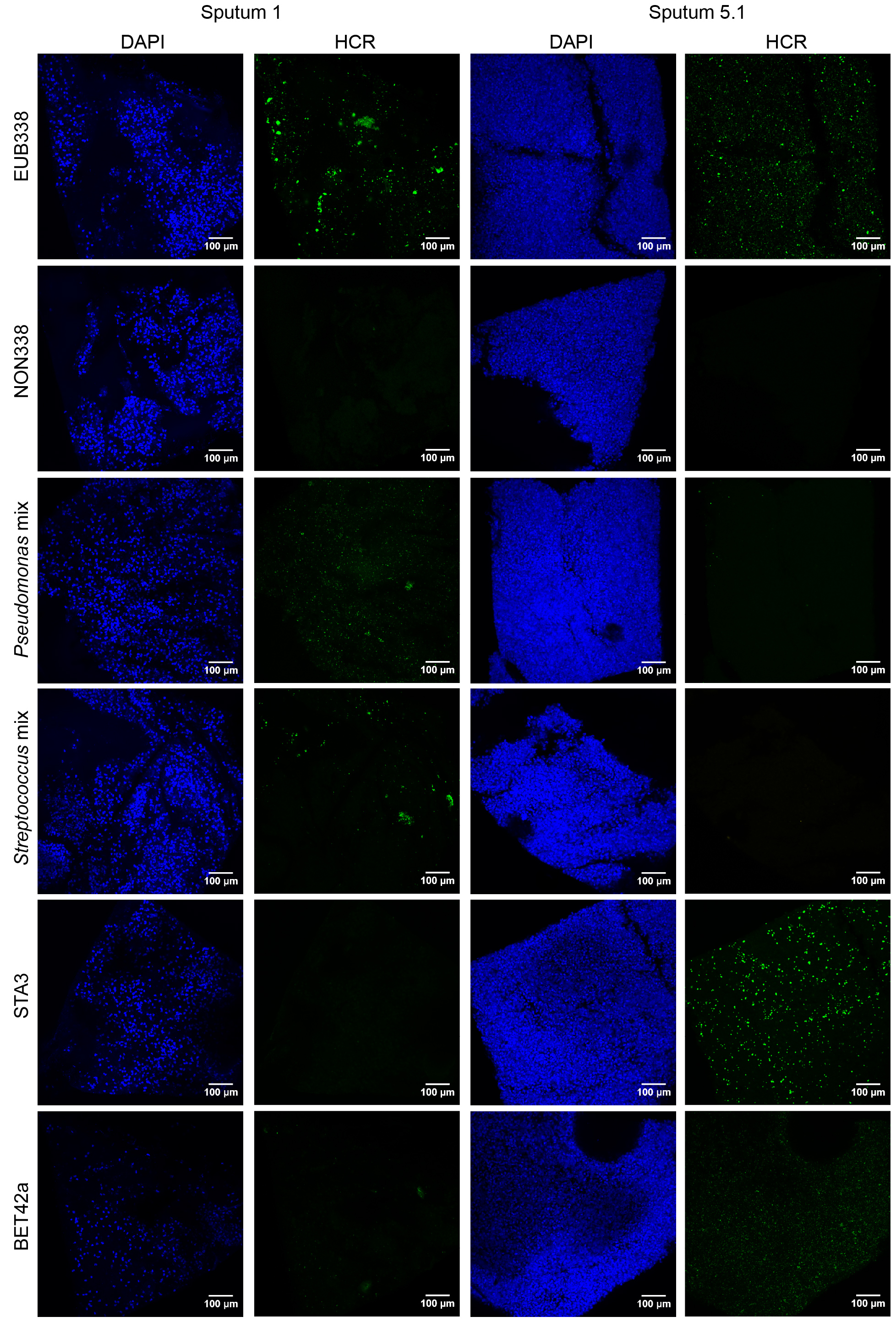

Supplement: Figure S5 — Cross-reactivity of HCR probes. HCR was performed on sputum samples 1 and 5.1 with the indicated probes/probe mixes and stained with DAPI. The panels depict 10-plane maximum intensity projections acquired with a 10× objective. EUB338-B4 was used with hairpin conjugated to AlexaFluor 647. NON338-B4 was used with hairpin conjugated to AlexaFluor 647. The Pseudomonas mix-B4 was used with hairpin conjugated to AlexaFluor 594. The Streptococcus probe mix-B4 was used with hairpin conjugated to AlexaFluor 594. STA3-B3 was used with hairpin conjugated to AlexaFluor 488. BET42a-B4 was used with hairpin conjugated to AlexaFluor 594. All bacteria are shown in green, and the results of DAPI staining (blue) are included to show sputum architecture. Laser power/gain settings were kept constant for both images acquired with a particular probe set (for NON338, the same laser power/gain settings as those for EUB338 were used). Download [file mbo004162984sf5.jpg]

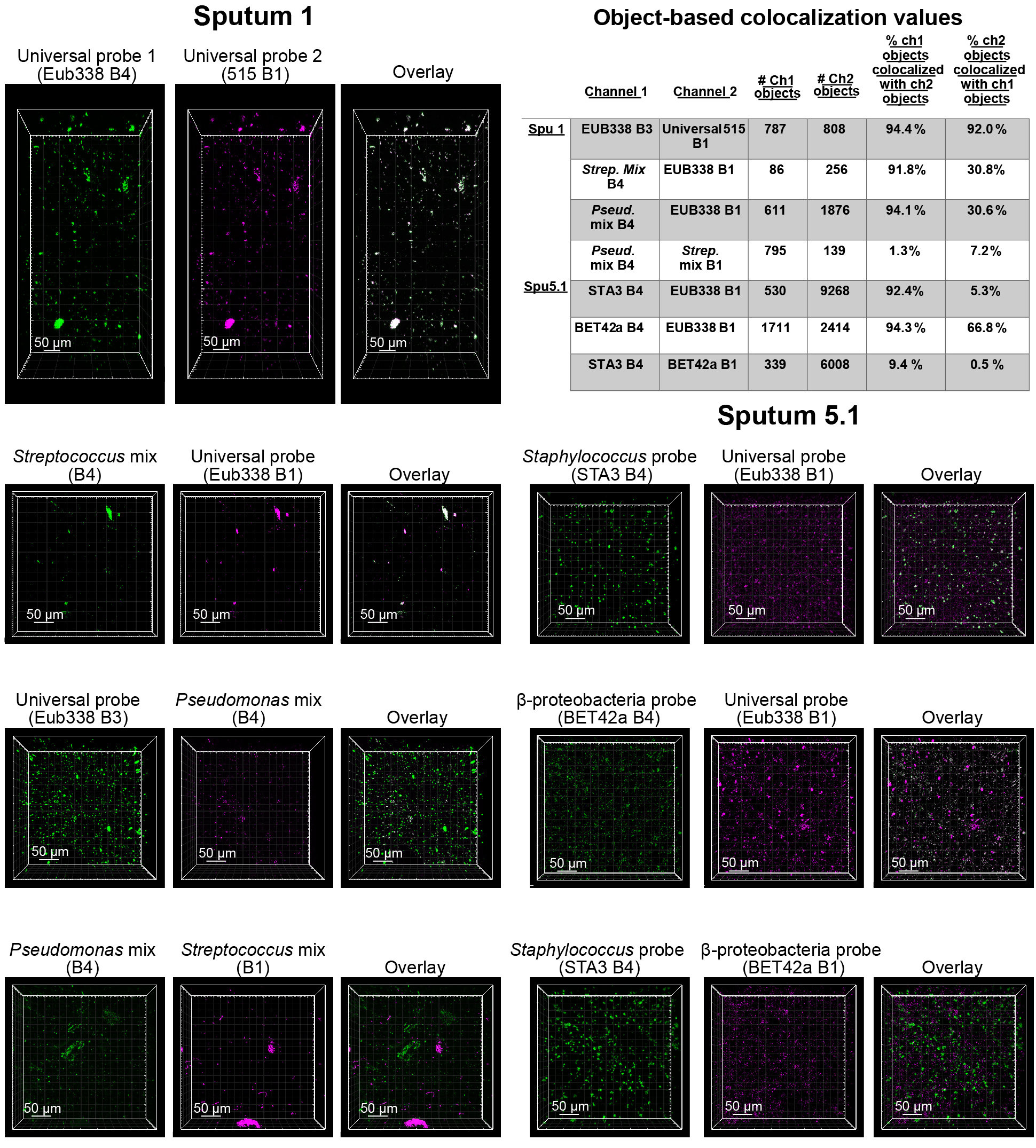

Supplement: Figure S6 — Object-based colocalization in sputum with HCR. Multiplex HCR was performed on sputum 1 or sputum 5.1 with two concurrent initiator probes (denoted in the figure). Both Str and Str56 were used for Streptococcus (Streptococcus mix), and PseaerA, PseaerB, Pae997, and PSE227 were used for Pseudomonas (Pseudomonas mix). Amplification was performed with one initiator probe set conjugated to AlexaFluor 488 (green) and one conjugated to AlexaFluor 647 (magenta). Z-stacks were acquired with a 25× objective, and maximum intensity projections are shown. Object-based colocalization was performed by calculating the percentage of objects in each channel that overlapped with objects in the other channel. Values are listed in table in the upper right panel. Download [file mbo004162984sf6.jpg]

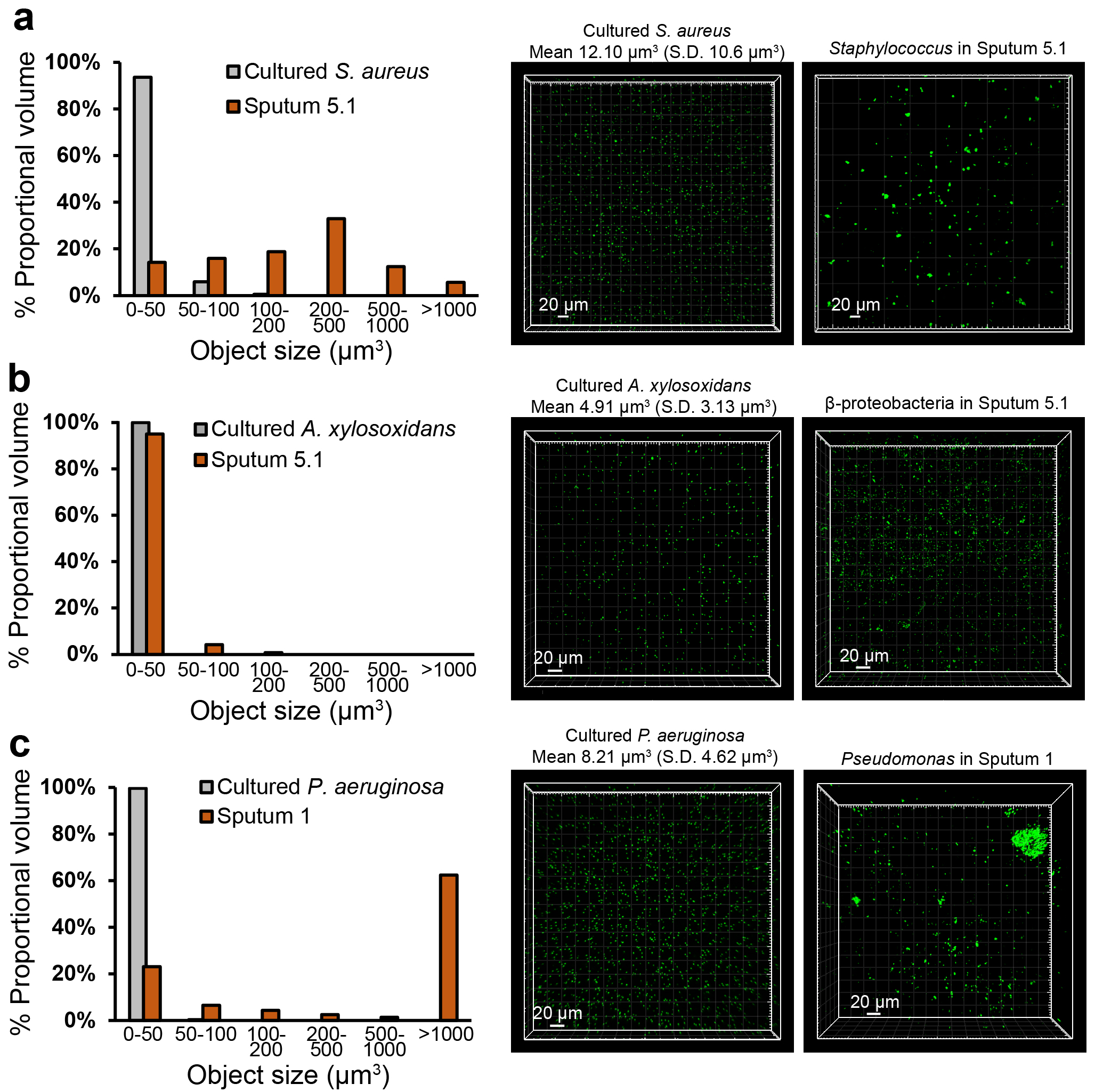

Supplement: Figure S7 — Size distribution of sputum aggregates compared to cultured cells. HCR was performed on cultured stationary-phase cells after fixing and embedding in acrylamide-based hydrogel blocks. Z-stacks were acquired with a 25× objective, and objects were binned according to proportional size (relative to the total fluorescent HCR volume of a sample). (A) Cultured stationary-phase S. aureus cells were compared to objects identified in sputum 5.1 with the Staphylococcus-probe mixture B4 with hairpins conjugated to AlexaFluor 647 (1,223 sputum objects analyzed). The Staphylococcus in sputum 5.1 image shows one panel out of four that were imaged and analyzed to obtain the histogram. (B) Cultured A. xylosoxidans harvested at stationary phase was compared to objects identified in sputum 5.1 with BET42a-B4 and hairpins conjugated to AlexaFluor 488 (4,091 sputum objects analyzed). (C) Cultured, stationary-phase P. aeruginosa was compared to objects identified in sputum 1 with a Pseudomonas-specific probe mixture and hairpins conjugated to AlexaFluor 647 (1,110 sputum objects analyzed). Download [file mbo004162984sf7.jpg]

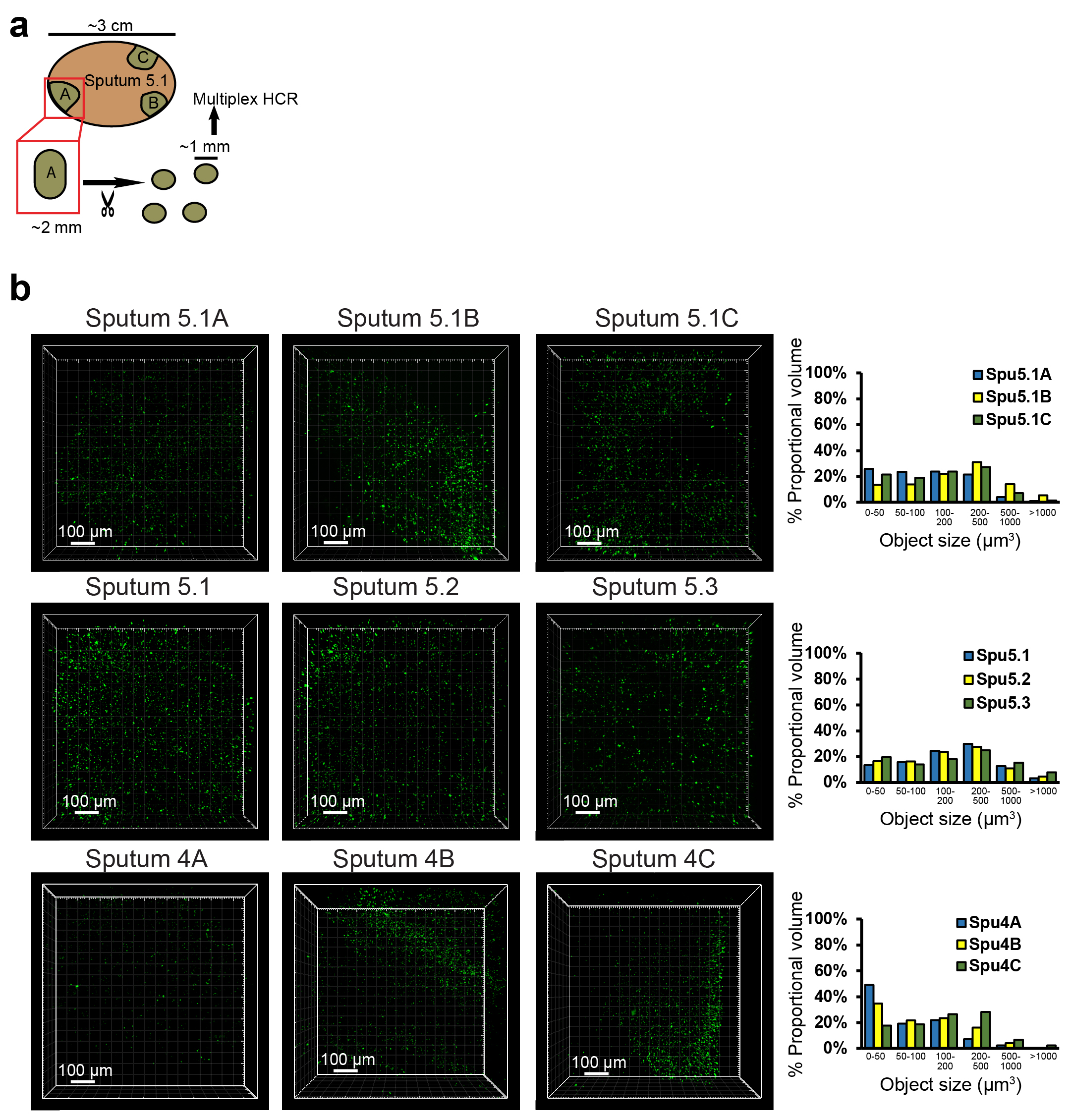

Supplement: Figure S8 — Intersample and interpatient S. aureus aggregation patterns (A) Diagram demonstrating how sputum samples were processed for confocal microscopy analysis. (B) Three different sections of sputum 5.1 were probed with Staphylococcus-specific probe STA3 with a B4 amplifier and B4 hairpins conjugated to AlexaFluor 647: 5.1A1 (2,852 objects analyzed), 5.1B1 (2,892 objects analyzed), and 5.1C1 (1,992 objects analyzed). Maximum intensity projections are shown. As in Fig. 2, objects were binned according to proportional size and histograms were graphed. The same analysis was performed on three samples collected at three different time points from patient 5, (STA3 with B4 amplifier and AlexaFluor 647 conjugated B4 hairpins): 5.1 (4,250 objects analyzed), 5.2 (103 days after 5.1; 3,714 objects analyzed), and 5.3 (1 day after 5.2; 2,574 objects analyzed), and on three separate sections of a sputum sample from patient 4 (STA3 with B4 amplifier and AlexaFluor 594-conjugated B4 hairpins): 4A (825 objects analyzed), 4B (4,538 objects analyzed), and 4C (3,043 objects analyzed). All Z-stacks were acquired with a 10× objective. Download [file mbo004162984sf8.jpg]

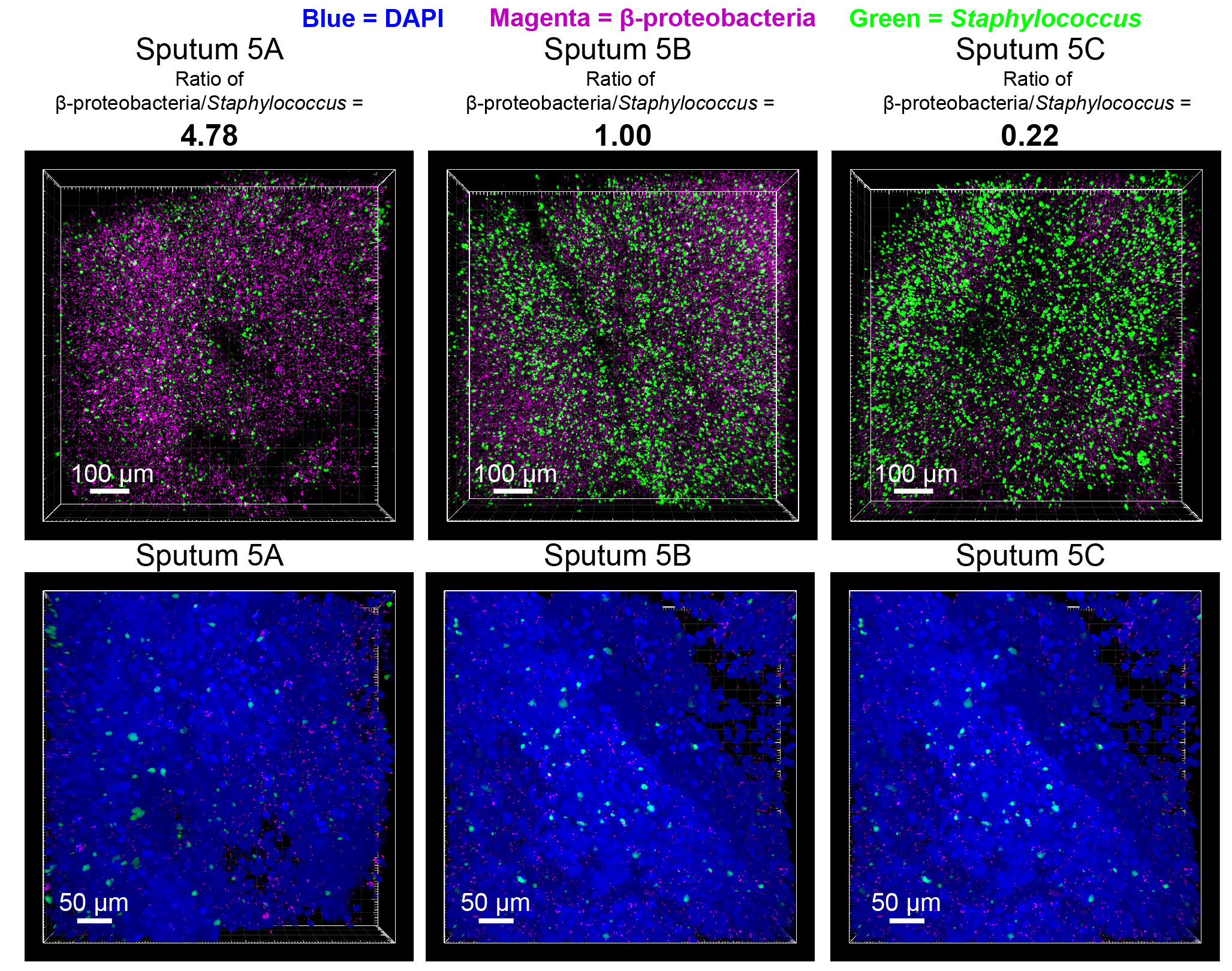

Supplement: Figure S9 — HCR multiplexing shows heterogeneity in the ratios of Betaproteobacteria to Staphylococcus in sputum sample 5.1. HCR with BET42a-B1 and hairpins conjugated to AlexaFluor 647 and STA3-B4 and hairpins conjugated to AlexaFluor 488 was performed on three separate regions of sputum sample 5.1. Z-stacks at 10× were acquired, and maximum intensity projections were produced (top row). To determine relative abundances of Staphylococcus and Betaproteobacteria in each section of sputum 5.1, the total fluorescence volume from the BET42a signal was divided by the total fluorescence volume from the STA3 signal. After DAPI staining, 25× Z-stacks were acquired, and blend projections were produced (bottom row). Download [file mbo004162984sf9.jpg]

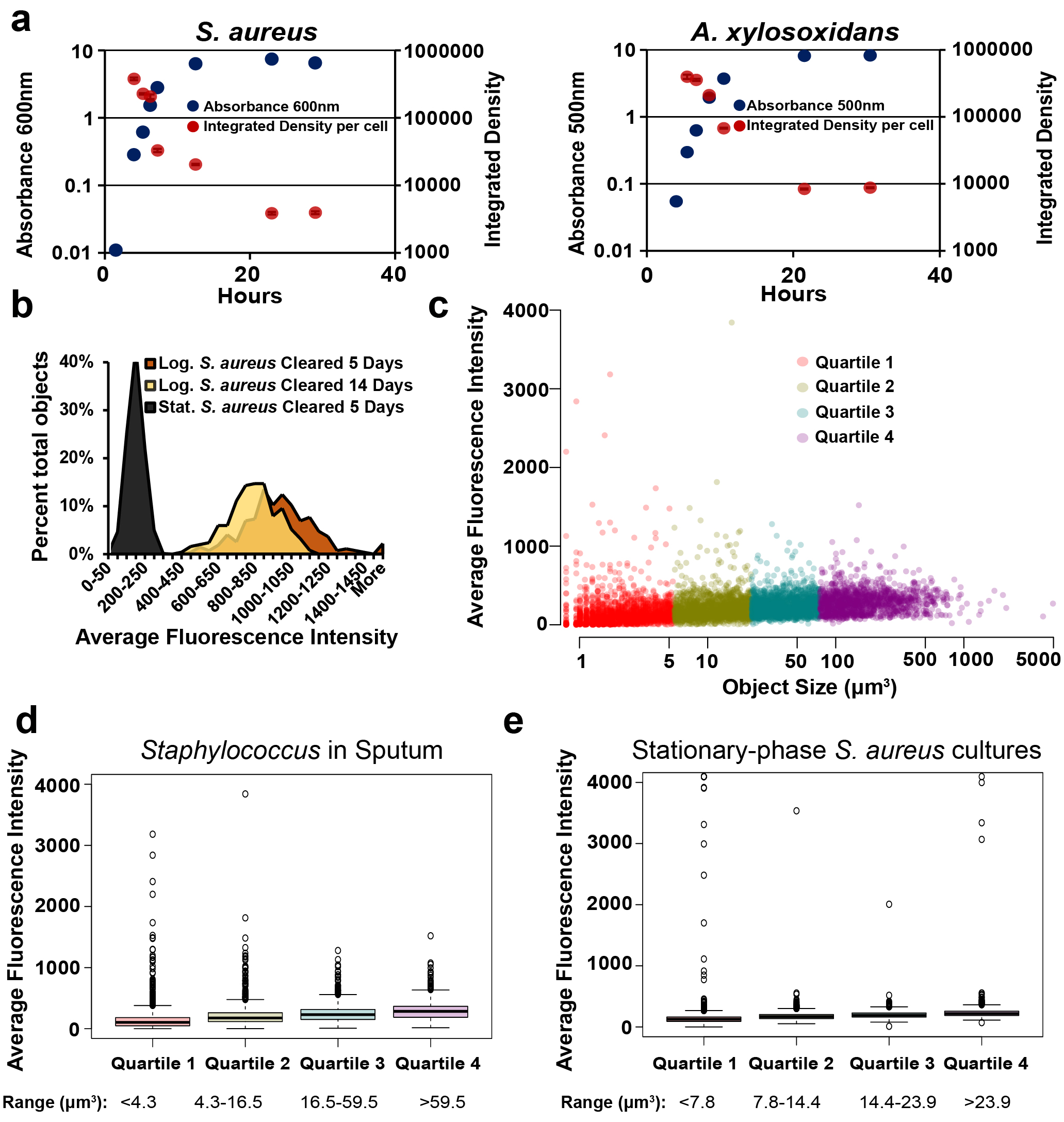

Supplement: Figure S10 — Growth rate controls and analysis. (A) Optical density of S. aureus and A. xylosoxidans cultures for growth curves plotted with the integrated density of cell fluorescence obtained by FISH with a EUB338 probe dilabeled with Cy3 from culture samples taken at each point along the growth curve. Error bars represent standard errors. For A. xylosoxidans, sample sizes from the earliest time point (5.5 h) to the last time point (30.5 h) were 78, 150, 465, 1,276, 1,504, and 1,303 cells, respectively. For S. aureus, sample sizes from the earliest time point (4 h) to the last time point (29 h) were 318, 954, 795, 1,139, 2,738, 395, and 193 cells, respectively. (B) Average fluorescence intensity shown for S. aureus logarithmic cultures cleared for 5 days (used as the standard in growth rate experiments) and 14 days, compared to S. aureus stationary-phase cultures cleared for 5 days (used as the standard in growth rate experiments). Growth rate measurements were performed as described for Fig. 4, with a STA3 HCR probe and hairpins conjugated to AlexaFluor 488, and a EUB338 FISH probe conjugated to Cy5. (C) All 6,757 STA3-positive objects that were analyzed from sputum samples 4, 5.1, 5.2, and 5.3 (Fig. 4) were broken up into quartiles by object size and graphed on a scatterplot of object size versus average fluorescence intensity. Box plots show average fluorescence intensity of the four quartiles for sputum samples (D) and for stationary-phase cultured S. aureus cells (E). A Welch’s two sample t test was applied to each adjacent quartile pair for both plots. Quartile 2 had a significantly increased average fluorescent intensity compared to quartile 1 (P < 2.2e−16 [D]; P = 1.2e−5 [E]), quartile 3 had a significantly increased average fluorescent intensity compared to quartile 2 (P < 1.2e−13 [D], P < 2.2e−16 [E]), and quartile 4 had a significantly increased average fluorescent intensity compared to quartile 3 (P < 2.2e−16 [D]; P < 2.2e−16 [E]). Plotting and analyses for the res [file mbo004162984sf10.jpg]
